# Beta-Band Resonance and Intrinsic Oscillations in a Biophysically Detailed Model of the Subthalamic Nucleus-Globus Pallidus Network

**DOI:** 10.3389/fncom.2019.00077

**Published:** 2019-11-05

**Authors:** Lucas A. Koelman, Madeleine M. Lowery

**Affiliations:** Neuromuscular Systems Laboratory, School of Electrical and Electronic Engineering, University College Dublin, Dublin, Ireland

**Keywords:** basal ganglia, subthalamic nucleus, Parkinson's disease, beta-band oscillations, synchronization, globus pallidus, multi-compartmental neuron model

## Abstract

Increased beta-band oscillatory activity in the basal ganglia network is associated with Parkinsonian motor symptoms and is suppressed with medication and deep brain stimulation (DBS). The origins of the beta-band oscillations, however, remains unclear with both intrinsic oscillations arising within the subthalamic nucleus (STN)—external globus pallidus (GPe) network and exogenous beta-activity, originating outside the network, proposed as potential sources of the pathological activity. The aim of this study was to explore the relative contribution of autonomous oscillations and exogenous oscillatory inputs in the generation of pathological oscillatory activity in a biophysically detailed model of the parkinsonian STN-GPe network. The network model accounts for the integration of synaptic currents and their interaction with intrinsic membrane currents in dendritic structures within the STN and GPe. The model was used to investigate the development of beta-band synchrony and bursting within the STN-GPe network by changing the balance of excitation and inhibition in both nuclei, and by adding exogenous oscillatory inputs with varying phase relationships through the hyperdirect cortico-subthalamic and indirect striato-pallidal pathways. The model showed an intrinsic susceptibility to beta-band oscillations that was manifest in weak autonomously generated oscillations within the STN-GPe network and in selective amplification of exogenous beta-band synaptic inputs near the network's endogenous oscillation frequency. The frequency at which this resonance peak occurred was determined by the net level of excitatory drive to the network. Intrinsic or endogenously generated oscillations were too weak to support a pacemaker role for the STN-GPe network, however, they were considerably amplified by sparse cortical beta inputs and were further amplified by striatal beta inputs that promoted anti-phase firing of the cortex and GPe, resulting in maximum transient inhibition of STN neurons. The model elucidates a mechanism of cortical patterning of the STN-GPe network through feedback inhibition whereby intrinsic susceptibility to beta-band oscillations can lead to phase locked spiking under parkinsonian conditions. These results point to resonance of endogenous oscillations with exogenous patterning of the STN-GPe network as a mechanism of pathological synchronization, and a role for the pallido-striatal feedback loop in amplifying beta oscillations.

## Introduction

Pathological oscillations in the basal ganglia-thalamocortical (BGTC) network have long been implicated in the motor symptoms of Parkinson's disease. Beta-band (13–30 Hz) oscillations are consistently strengthened with dopamine depletion both in individuals with Parkinson's disease (PD) and parkinsonian animal models (Sharott et al., 2005; Kuhn et al., 2008; Mallet et al., 2008b), and are reduced by deep brain stimulation (DBS) and pharmacological interventions that alleviate parkinsonian motor symptoms (Kühn et al., 2006; Weinberger et al., 2006; Ray et al., 2008; Eusebio et al., 2011). The magnitude of subthalamic nucleus local field potential beta oscillations is also correlated with the severity and degree of improvement of bradykinetic/akinetic motor symptoms and rigidity (Kühn et al., 2006; Bronte-Stewart et al., 2009). Although beta-band oscillations may not be causal to bradykinetic/akinetic symptoms (Leblois et al., 2007), they offer potential as a biomarker for symptom severity and the underlying network pathophysiology in advanced Parkinson's Disease. The origin of beta-band oscillations in the BGTC network, however, remains unclear. The most prominent hypotheses emphasize the importance of dopamine-modulated strengthening of particular feedback loops within the BGTC network. Computational models have provided a valuable tool with which to explore various hypotheses regarding the mechanisms by which oscillatory activity with the network is generated. Different models have placed the origin of beta and sub-beta band oscillations in the STN-GPe network (Terman et al., 2002; Gillies and Willshaw, 2007; Holgado et al., 2010; Pavlides et al., 2012), in cortical and thalamo-cortical circuits (Pavlides et al., 2015; Sherman et al., 2016; Liu et al., 2017; Reis et al., 2019), in striatal or pallidostriatal circuits (McCarthy et al., 2011; Corbit et al., 2016), or in the full BGTC loop (Leblois, 2006; Kang and Lowery, 2013; Pavlides et al., 2015; Kumaravelu et al., 2016). These models show that under many conditions the network is prone to oscillate, through intrinsic pacemaking or susceptibility to an extrinsic rhythm.

The reciprocally connected subthalamo-pallidal (STN-GPe) network is a key site in the basal ganglia in which beta-band oscillations are manifest in Parkinson's disease (Mallet et al., 2008a,b). This network was an early focus of modeling studies due to its reciprocally connected structure and ability to generate low frequency oscillations in tissue cultures (Plenz and Kital, 1999). Models of the STN-GPe as a pacemaker initially focused on the generation of low frequency oscillations within the frequency range of parkinsonian tremor (Gillies et al., 2002; Terman et al., 2002), with focus shifting to the beta-band with increasing evidence of a link between beta activity and parkinsonian motor symptoms (Holgado et al., 2010; Pavlides et al., 2012).

More recent experimental evidence suggests that, rather than the STN-GPe network operating in a pacemaking mode, patterning by cortex may play a critical role in the generation of pathological beta-band oscillations in Parkinson's disease. This is supported by observations of high functional coupling between cortex and STN (Magill et al., 2004; Sharott et al., 2005; Mallet et al., 2008a; Litvak et al., 2011; Moran et al., 2011), and that oscillatory activity in STN-GPe is contingent on inputs from the cortex and can be abolished by disrupting them (Magill et al., 2001; Drouot et al., 2004; Tachibana et al., 2011). Cortical patterning of the STN-GPe network by means of feedback inhibition provides a proposed mechanism for this functional coupling (Baufreton et al., 2005; Bevan et al., 2006; Mallet et al., 2008a, 2012; Tachibana et al., 2011). According to this hypothesis, weak oscillatory activity arriving via cortico-STN afferents is amplified in the STN-GPe network when feedback inhibition from the GPe is offset in phase with cortical excitation. While such feedback-mediated oscillations have been observed *in vivo* (Paz, 2005) and in slices (Baufreton et al., 2005), the ability of the network to generate autonomous oscillations and its resonant response properties are still poorly understood. Specifically, it is not clear whether the STN-GPe network plays an active part in generating beta-band oscillations, nor whether it amplifies or merely sustains them. Neither is it fully understood how beta-band oscillations relate to other pathological patterns of neural activity in the subthalamic nucleus (STN) and external globus pallidus (GPe) that correlate more strongly with parkinsonian motor symptoms, notably increased neural bursting (Sanders et al., 2013; Sharott et al., 2014). It is clear, however, that interventions in the loop and its afferents that reduce beta-band oscillations (Tachibana et al., 2011) or bursting (Gradinaru et al., 2009; Pan et al., 2016; Sanders and Jaeger, 2016) lead to improvements in motor symptoms. Similarly, the STN (Benabid et al., 2009) and GPe, in non-human primates (Vitek et al., 2012), are effective targets for DBS.

Previous modeling studies have focused on alterations in connection patterns and strength within or between nuclei, typically represented by mean-field or single-compartment spiking neuron models. While such models are computationally efficient, they may not fully capture the role of intrinsic properties of neurons in shaping pathological activity patterns. Although cell-specific ion channels can be used, single-compartment neuron models lump together ion channels and synapses in one isopotential compartment in a way that may not capture the complex dynamics that arise when non-uniformly distributed ion channels (Gillies and Willshaw, 2005) interact with synapses associated with distinct subcellular regions (Bevan et al., 1995; Galvan et al., 2004; Pan et al., 2016). Hence they may not fully account for the mechanisms contributing to pathological activity within the STN and the role that synaptic-ionic current interactions play in sustaining beta-band oscillations and excessive burst firing.

It has recently been demonstrated that following dopamine depletion the balance of excitatory and inhibitory synaptic currents in STN neurons is shifted toward inhibition (Chu et al., 2017; Wang et al., 2018), known to promote burst responses by increasing the availability of Ca^2+^ and Na^+^ channels de-inactivated at hyperpolarized potentials (Baufreton et al., 2005). In the GPe increased inhibition, caused mainly by strengthening of striato-pallidal afferents, is also believed to play a role in generating pathological oscillations as demonstrated in model simulation (Gillies et al., 2002; Terman et al., 2002; Holgado et al., 2010; Kumar et al., 2011). Increased GPe inhibition has been suggested to cause increased engagement of HCN channels (Chan, 2004), which are involved in phase resetting and controlling the regularity of firing. However, whether functional coupling between BG nuclei is also moderated by the excitation-inhibition balance is not fully understood.

The aim of this study was, therefore, to examine the relative contributions of intrinsic, endogenously generated oscillations and patterning by exogenous oscillatory inputs in the generation of synchronous beta-band oscillatory activity in a biophysically detailed model of the parkinsonian STN-GPe network and the underlying biophysical mechanisms. A second aim was to understand how pathological oscillations and bursting patterns are related to the balance of excitation and inhibition in the STN and GPe. The STN-GPe network was modeled using biophysically detailed multi-compartmental cell models of STN and GPe neurons that capture the interaction between synaptic and intrinsic currents distributed within the dendritic structure and involved in autonomous pacemaking and bursting (Gillies and Willshaw, 2005; Gunay et al., 2008). The generation of oscillations both autonomously within the network and in response to beta frequency inputs from the cortex (CTX) and indirect pathway striatal medium spiny neurons (iMSN) was examined as the balance of excitation and inhibition within the network was systematically varied, and oscillatory inputs with varying phase relationships were added. A better understanding of the relative contribution of these different factors and their interaction has the potential to improve understanding of the mechanism of action of existing anti-parkinsonian therapies, including DBS and to guide the development of more effective circuit interventions.

## Methods

### Model Architecture

**Figure 1:**
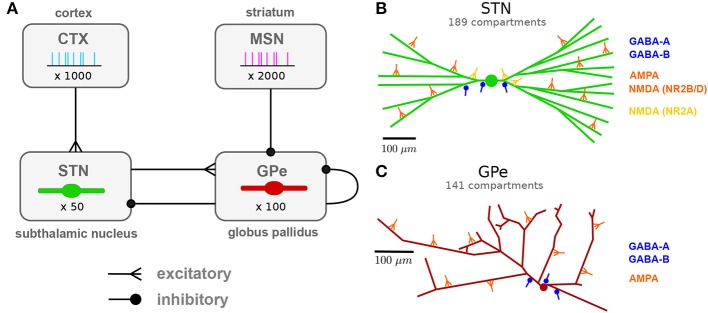
Network architecture: population and subcellular connectivity. **(A)** Neuronal populations and their projections modeled in the network. Subthalamic projection neurons (STN) and prototypic neurons of the external globus pallidus (GPe) were modeled using multi-compartmental neuron models. Cortical projection neurons (CTX) and indirect pathway striatal medium spiny neurons (iMSN) were modeled as spike generators. **(B)** Branching structure of the STN neuron model and representative synapses by afferent type, indicating subcellular distribution of synapses. Cortical glutamergic afferents synapse primarily onto thin dendrites, distally relative to the soma, but NMDA receptors with faster NR2A subunits mainly target the soma and proximal areas. Pallidal GABAergic afferents target proximal areas of the cell. **(C)** Branching structure of the GPe neuron model and representative synapses by afferent type. GABAergic GPe-GPe collaterals mainly target somata and proximal dendrites, whereas glutamergic afferents were placed in distal regions. Full details of the model are provided in the section Methods.

The network model of the STN-GPe network consisted of four populations of neurons (Figure 1): the STN and GPe neurons, modeled as multi-compartmental conductance-based models, and their cortical and striatal inputs, modeled as Poisson or bursting spike generators.

Population sizes were chosen to preserve the decrease in population sizes and convergence of projections along the indirect and hyperdirect pathways in the basal ganglia. The STN and GPe populations consisted of 50 and 100 multi-compartmental cells, respectively, to approximate the ratio of 13,000 STN cells to 30,000 GPe prototypic cells (Oorschot et al., 1999; Abdi et al., 2015) unilaterally in the rat. As a source of synaptic noise, an additional 10% of the cells in the STN and GPe populations were modeled as Poisson spike generators firing at a mean rate equal to the experimentally reported rate for the modeled state.

The cortical and striatal populations consisted of 1,000 and 2,000 cells, respectively, modeled as spike generators. These numbers were chosen to have 20 independent pre-synaptic spike generators per post-synaptic cell to model convergence along the hyperdirect CTX-STN and indirect iMSN-GPe projection. For the iMSN-GPe projection, convergence from all medium spiny neurons (MSN) to GPe, ignoring subpopulations, is 2,800,000 MSN cells to 46,000 GPe cells (Oorschot, 1996) resulting in a convergence factor of 60. Assuming that convergence is similar between iMSN and GPe prototypic neurons, our number is an underestimation by a factor three. Because iMSN cells in our model spike independently and since the number of synapses per cell was lower than in reality, this was considered acceptable.

Stochastic connectivity profiles for the connections illustrated in Figure 1 were generated by randomly selecting a fixed number of afferents from the pre-synaptic population for each post-synaptic cell. The ratios of number of afferents from each source population were determined, where possible, based on the reported number of synaptic boutons per afferent type and the number of contacts per axon (Table 1). Each multi-synaptic contact was represented by a single synapse to reduce the number of simulated synapses to a more tractable number.

**Table 1:** Experimentally reported connection parameters used to calibrate the model.

**Target**	**Source**	**Afferent neurons**	**Synaptic contacts**	**Subcellular targets**	**Short-term plasticity**	**Delay**	**Effect of dopamine depletion**
STN	(all)	300 (Baufreton and Bevan, 2008)		N.A.	N.A.	N.A.	N.A.
	CTX			distal (Bevan et al., 1995; Mathai et al., 2015; Pan et al., 2016),	depression (Chu et al., 2015)	5.9 ms (Kita and Kita, 2011)	weakened (Chu et al., 2017; Wang et al., 2018)
				proximal (Pan et al., 2016)			
	GPe	57 (Atherton et al., 2013)	883 (Baufreton et al., 2009)	proximal (Smith et al., 1990)	depression (Atherton et al., 2013)	4 ms (Fujimoto and Kita, 1993)	strengthened (Chu et al., 2015)
							prolonged decay (Fan et al., 2012)
GPe	GPe			proximal,somatic (Chan, 2004; Sadek et al., 2007)	depression (Miguelez et al., 2012)		strengthened (Miguelez et al., 2012)
	STN	135 (Kita and Jaeger, 2016)		dendritic,distal (Shink and Smith, 1995)	facilitation,	2 ms (Kita and Kitai, 1991)	strengthened (Hernández et al., 2006)
					depression (Hanson and Jaeger, 2002)		
	MSN		10622 (Kita and Jaeger, 2016)	dendritic, distal (Chan, 2004)	facilitation (Miguelez et al., 2012)	5 ms (Kita and Kitai, 1991)	

### Conductance-Based Models

The membrane potential *v*_*j*_ (mV) in each compartment *j* of a multi-compartmental cable model is governed by:

(1) $$\begin{array}{rc} c_{m}\frac{\delta v_{j}}{\delta t} & =\frac{d}{4R_{a}}\frac{\delta^{2}V}{\delta x^{2}}-g_{m}\left(V-E_{m}\right)-\sum I_{ion,j}-\sum I_{syn,j} \end{array}$$

where *x* (cm) is the position along the cable, *c*_*m*_ (μ*F*/*cm*^2^) is the specific membrane capacitance, *d* (cm) is the cable diameter, *R*_*a*_ is the specific axial resistance (Ω*cm*), *g*_*m*_ (*S*/*cm*^2^) is the passive membrane conductance, *E*_*m*_ (mV) the leakage reversal potential, *I*_*ion, j*_ (*mA*/*cm*^2^) are the ionic currents flowing across the membrane of compartment *j*, and *I*_*syn, j*_ (*mA*/*cm*^2^) are the synaptic currents at synapses placed in the compartment. Each ionic current is governed by an equation of the form:

(2) $$\begin{array}{r} I_{x}={\bar{g}}_{x}m_{x}^{p}h_{x}^{q}\left(V-E_{x}\right) \end{array}$$

where ${\bar{g}}_{x}$ is the maximum conductance of the channel (*S*/*cm*^2^), *E*_*x*_ is the reversal potential (mV), and *m*_*x*_ and *h*_*x*_ the open fractions of the activation and inactivation gates. The dynamics of the activation and inactivation gates *m* and *h* are governed by

(3) $$\begin{array}{r} \frac{dm}{dt}=\frac{m_{\infty }\left(v\right)-m}{\tau_{m}\left(v\right)}, \end{array}$$

with *m*_∞_(*v*) and τ_*m*_(*v*) representing the voltage-dependent steady state value and time constant of the gate. For some currents the gating dynamics are described in terms of the opening and closing rates α_*m*_ and β_*m*_ related through $\tau_{m}=\frac{1}{\alpha_{m}+\beta_{m}}$, $m_{\infty }=\frac{\alpha_{m}}{\alpha_{m}+\beta_{m}}$ :

(4) $$\begin{array}{r} \frac{dm}{dt}=\alpha_{m}\left(v\right)\cdot \left(1-m\right)-\beta_{m}\left(v\right)\cdot m. \end{array}$$

Reversal potentials are assumed constant unless otherwise noted. The reversal potential for *Ca*^2+^ currents was calculated using the Nernst equation from the intra- and extracellular ion concentrations:

(5) $$\begin{array}{r} E_{Ca}=\frac{RT}{zF}\ln\;\frac{{\left[{Ca}^{2+}\right]}_{o}}{{\left[{Ca}^{2+}\right]}_{i}} \end{array}$$

where *T* is the temperature in Kelvin, *R* is the universal gas constant, *F* is the Faraday constant, and *z* is the valence of the calcium ion (+2). Intracellular calcium buffering in a sub-membrane shell is modeled as:

(6) $$\begin{array}{rc} \frac{d{\left[{Ca}^{2+}\right]}_{i}}{dt} & =-\left(I_{\text{CaL}}+I_{\text{CaN}}+I_{\text{CaT}}\right)\frac{c}{2Fd}-\frac{{\left[{Ca}^{2+}\right]}_{i0}-{\left[{Ca}^{2+}\right]}_{i}}{\tau_{\text{Ca}}} \end{array}$$

where *c* is a unit conversion constant, *d* is the thickness of the sub-membrane shell, and τ_Ca_ is the time constant of decay.

Synaptic connections between cells were modeled by spike detectors in the somatic compartments, coupled to synapses in the target cells by a time delay. As no interactions between axons and other biophysical processes such as electric fields were required, axonal structures were omitted from the model and represented as delays between connected neurons. This constrained the computational complexity of the model, avoiding the requirement to simulate large number of additional compartments without altering the network behavior. Synapses were modeled by a dual exponential profile with rise and decay times τ_*rise*_ and τ_*decay*_ modulated by the fraction of synaptic resources in the active state which was governed by Tsodyks-Markram dynamics (Tsodyks et al., 1998):

(7) $$\begin{array}{r} I_{syn}={\bar{g}}_{syn}\left(B-A\right)\left(v-E_{syn}\right) \end{array}$$

(8) $$\begin{array}{rc} \frac{dA}{dt} & =\frac{-A}{\tau_{rise}}+f_{peak}\cdot U_{SE}\cdot R\cdot \delta \left(t-t_{spk}\right) \end{array}$$

(9) $$\begin{array}{rc} \frac{dB}{dt} & =\frac{-B}{\tau_{decay}}+f_{peak}\cdot U_{SE}\cdot R\cdot \delta \left(t-t_{spk}\right) \end{array}$$

(10) $$\begin{array}{rc} \frac{dR}{dt} & =\frac{1-R}{\tau_{rec}}-U_{SE}\cdot R\cdot \delta \left(t-t_{spk}\right) \end{array}$$

(11) $$\begin{array}{rc} \frac{dU_{SE}}{dt} & =\frac{-U_{SE}}{\tau_{facil}}+U_{1}\cdot \left(1-U_{SE}\right)\cdot \delta \left(t-t_{spk}\right) \end{array}$$

(12) $$\begin{array}{rc} f_{peak} & =\frac{1}{\exp\left(-t_{peak}/\tau_{decay}\right)-\exp\left(-t_{peak}/\tau_{rise}\right)} \end{array}$$

(13) $$\begin{array}{rc} t_{peak} & =\frac{\tau_{rise}\cdot \tau_{decay}}{\tau_{decay}-\tau_{rise}}\log\left(\frac{\tau_{decay}}{\tau_{rise}}\right) \end{array}$$

where, ${\bar{g}}_{syn}$ is the peak synaptic conductance, B-A represents the synaptic gating variable, *f*_*peak*_ is a normalization factor so that B-A reaches its maximum at time *t*_*peak*_ after the time of spike arrival *t*_*spk*_, R is the fraction of vesicles available for release, *U*_*SE*_ is the release probability, and τ_*rec*_ and τ_*facil*_ are the time constants for recovery from short-term depression and facilitation, respectively. The synaptic reversal potentials *E*_*syn*_ were 0 mV for AMPA and NMDA, −80 mV for GABA_A_, and −95 mV for GABA_B_. For NMDA synapses there is an additional voltage-dependent gating variable representing magnesium block (Jahr and Stevens, 1990):

(14) $$\begin{array}{r} m\left(v\right)=1/\left(1+exp\left(-0.062v\right)*\left(1/3.57\right)\right) \end{array}$$

The metabotropoc GABA_B_ receptor-mediated current was modeled as an intracellular signaling cascade based on the model by Destexhe and Sejnowski (1995). The equations describing G-protein activation and the synaptic current were retained, but the bound receptor fraction including the effects of desensitization was represented by the fraction of resources in the active state in the Tsodyks-Markram scheme (B-A). The equation governing the G-protein production rate thus became

(15) $$\begin{array}{r} \frac{dG}{dt}=K3*\left(B-A\right)-K4*G \end{array}$$

where G is the G-protein concentration, and K3 and K4 are the rates of G-protein production and decay, respectively. The G-protein concentration *G* gates the peak synaptic conductance according to a sigmoid activation function represented by the Hill equation:

(16) $$\begin{array}{r} I_{\text{GAB}{\text{A}}_{\text{B}}}={\bar{g}}_{syn}\frac{G^{n}}{G^{n}+K_{d}^{n}}\left(v-E_{GABA_{B}}\right). \end{array}$$

### STN Cell Model

STN neurons were modeled using the rat subthalamic projection neuron model by Gillies and Willshaw (2005) (ModelDB accession number 74298). The neuron morphology is based on quantitative characterization of the dendritic trees of STN neurons *in vitro*. The model includes 10 intrinsic ionic currents (Table 2) :

(17) $$\begin{array}{l} I_{ion,j}=I_{NaF}+I_{NaP}\\ \;+I_{KDR}+I_{Kv31}+I_{sKCa}\\ \;+I_{CaT}+I_{CaL}+I_{CaN}\\ \;+I_{HCN}+I_{L} \end{array}$$

where *I*_*NaF*_ and *I*_*NaP*_ are the transient fast-acting and persistent sodium current, *I*_*KDR*_, *I*_*Kv*31_, and *I*_*sKCa*_ the delayed rectifier, fast rectifier and calcium-activated potassium current, *I*_*CaT*_, *I*_*CaL*_, and *I*_*CaN*_ the low-voltage-activated T-type, high-voltage-activated L-type, and high-voltage-activated N-type calcium currents, *I*_*HCN*_ the hyperpolarization-activated cyclic nucleotide (HCN) current, and *I*_*L*_ the leak current. The equations governing the dynamics of the gating variables are listed in Table 2. The channel density distributions are described extensively in Gillies and Willshaw (2005). As a source of noise, a current with a Gaussian amplitude distribution, mean zero and standard deviation 0.1 was added to the somatic compartment.

**Table 2:** STN model intrinsic current equations from Gillies and Willshaw (2005).

**Current**	**Equation**	**Gating variables**		**Parameters**
*I*_*NaF*_	${\bar{g}}_{NaF}m^{2}h\left(v-E_{Na}\right)$	$\alpha_{m}=0.32\frac{\left(13.1-v\right)}{\exp\left(\left(13.1-v\right)/4\right)-1}$	$\beta_{m}=0.28\frac{\left(v-40,1\right)}{\exp\left(v-40.1\right)-1}$	${\bar{g}}_{NaF}=14.83\text{e}-3$ (soma)
		$\alpha_{h}=0.128\exp\left(\frac{17-v}{18}\right)$	$\beta_{h}=\frac{4}{\exp\left(\left(40-v\right)/5\right)+1}$	${\bar{g}}_{NaF}=1\text{e}-7$ (dendrite)
*I*_*NaP*_	${\bar{g}}_{NaP}\left(v-E_{Na}\right)$			${\bar{g}}_{NaP}=1.11\text{e}-5$ (soma)
				${\bar{g}}_{NaP}=8.10\text{e}-6$ (dendrite)
*I*_*KDR*_	${\bar{g}}_{KDR}n\left(v-E_{K}\right)$	$\alpha_{n}=\frac{0.016\left(35.1-v\right)}{\exp\left(\left(35.1-v\right)/5\right)-1}$	β_*n*_ = 0.25exp((20−*v*)/40)	${\bar{g}}_{KDR}=3.84\text{e}-3$ (soma)
				${\bar{g}}_{KDR}\in \left[4.22,9.32\right]\times 10^{5}$ (dendrite)
*I*_*Kv*31_	${\bar{g}}_{Kv31}p\left(v-E_{K}\right)$	$p_{\infty }=\frac{1}{1+\exp\left(-\left(v+5\right)/9\right)}$	$\tau_{\infty }=\frac{18.71}{\exp\left(-\left(v+28\right)/6\right)+\exp\left(\left(v+4\right)/16\right)}$	${\bar{g}}_{Kv31}=1.34\text{e}-2$ (soma)
				${\bar{g}}_{Kv31}\in \left[8.91,10\right]\times 10^{4}$ (dendrite)
*I*_*sKCa*_	${\bar{g}}_{sKCa}w\left(v-E_{K}\right)$	$w_{\infty }=\frac{0.81}{1+\exp\frac{-\log{\left[Ca^{2+}\right]}_{i}-0.3}{0.46}}$	τ_*w*_ = 40	${\bar{g}}_{sKCa}=6.84\text{e}-5$ (soma)
				${\bar{g}}_{sKCa}=3.92\text{e}-5$ (dendrite)
*I*_*HCN*_	${\bar{g}}_{HCN}f\left(v-E_{HCN}\right)$	$f_{\infty }=\frac{1}{1+\exp\left(V+75\right)/5.5]}$	$\tau_{f}=\frac{1}{\exp\left(-14.59-.086v\right)+\exp\left(-1.87+.07v\right)}$	${\bar{g}}_{HCN}=1.01\text{e}-3$ (soma)
				${\bar{g}}_{HCN}=5.10\text{e}-4$ (dendrite)
*I*_*CaT*_	${\bar{g}}_{CaT}r^{3}s\left(v-E_{Ca}\right)$	$\alpha_{r}=\frac{1}{1.7+\exp\left(-\left(v+28.2\right)/13.5\right)}$	$\beta_{r}=\frac{\exp\left(-\left(v+63\right)/7.8\right)}{1.7+\exp\left(-\left(v+28.8\right)/13.5\right)}$	${\bar{g}}_{CaT}=0$ (soma)
		α_*s*_ = exp[−(*v*+160.3)/17.8]	$\beta_{s}=\left(\sqrt{.25+\exp\frac{v+83.5}{6.3}}-.5\right)k_{s}$	${\bar{g}}_{CaT}\in \left[1.17,1.67\right]\times 10^{3}$ (dendrite)
		$\alpha_{d}=\frac{1+\exp\left[\frac{\left(v+37.4\right)}{30}\right]}{240\left(0.5+\sqrt{0.25+\exp\left[\frac{\left(v+83.5\right)}{6.3}\right]}\right)}$	*k*_*s*_ = exp[−(*v*+160.3)/17.8]	${\left[{Ca}^{2+}\right]}_{i0}=1\text{e}-4$
			$\beta_{d}=\left(\sqrt{0.25+\exp\frac{v+83.5}{6.3}}-0.5\right)\alpha_{d}\left(v\right)$	τ_Ca_ = 185.7
*I*_*CaL*_	$g_{\text{CaT}}q^{2}h\left(v-E_{Ca}\right)$	$h_{\infty }\left({\left[{Ca}^{2+}\right]}_{i}\right)=0.53+\frac{0.47}{1+\exp\left(\frac{{\left[{Ca}^{2+}\right]}_{i}-0.7}{0.15}\right)}$	$\tau_{\infty }\left({\left[{Ca}^{2+}\right]}_{i}\right)=1220$	${\bar{g}}_{CaL}=9.50\text{e}-4$ (soma)
		$q_{\infty }\left(v\right)=\frac{1}{1+\exp\left[-\left(24.6v\right)/11.3\right]}$	$\tau_{q}\left(v\right)=\frac{1.25}{\cosh\left[-0.03\left(v+37.1\right)\right]}$	${\bar{g}}_{CaL}\in \left[1.21,18.7\right]\times 10^{4}$ (dendrite)
*I*_*CaN*_	$g_{\text{CaN}}q^{2}\left(v-E_{Ca}\right)$	$u_{\infty }\left(v_{j}\right)=\frac{1}{1+\exp\left[\left(v_{j}+60\right)/12.5\right]}$	τ_*u*_(*v*) = 98+cosh[0.021(10.1−*v*)]	${\bar{g}}_{CaN}=1.15\text{e}-3$ (soma)
				${\bar{g}}_{CaN}=4.79\text{e}-4$ (dendrite)

The synaptic currents included an excitatory glutamergic input from cortex, acting through AMPA and NMDA receptors, and an inhibitory GABAergic input from the GPe, acting through GABA_A_ and GABA_B_ receptors (Table 3):

(18) $$\begin{array}{l} I_{syn,j}=I_{\text{CTX}-\text{STN},\text{AMPA}}+I_{\text{CTX}-\text{STN},\text{NMDA}}\\ \;+I_{\text{GPE}-\text{STN},\text{GAB}{\text{A}}_{\text{A}}}+I_{\text{GPE}-\text{STN},\text{GAB}{\text{A}}_{\text{B}}} \end{array}$$

In the control condition STN neurons had 20 excitatory afferents from CTX neurons and 8 inhibitory afferents from GPe neurons. The location of synapses on STN neurons and axonal propagation delays were based on experimental observations (Table 1). Cortico-subthalamic (CTX-STN) synapses were modeled as conductance-based synapses with Tsodyks-Markram dynamics (Tsodyks et al., 1998). On each of its target cells, a cortical axon had one synapse located distally in the dendritic tree and one located proximally near the soma. Distal synapses had both an AMPA and slower NMDA conductance component. The latter represented slower-kinetics NMDA receptors with majority NR2B and NR2D subunits that have dendritic punctual expression (Pan et al., 2016). Proximal synapses had only an NMDA component and represented NMDA receptors with fast-kinetics NR2A subunits. Synaptic parameter values are listed in Table 3. Synaptic rise and decay time constants τ_*rise*_ and τ_*decay*_ for AMPA and NMDA NR2A constants were based on traces reported in Chu et al. (2015). For the slower NMDA NR2B synapses, values were based on Flint et al. (1997). The propagation delay *t*_*d*_ was taken from Kita and Kita (2011). Synapses were made to exhibit short-term depression upon high-frequency activation, based on observations by Froux et al. (2018). The ratio of the total AMPA to NMDA conductance were based on the ratios reported in Shen and Johnson (2005) for the normal and dopamine-depleted conditions, taking into account the reduction of synaptic terminals reported in Chu et al. (2017). Absolute values for the synaptic conductances were hand-tuned to bring the mean population firing rates into the reported range for the rat in dopamine-depleted condition (Mallet et al., 2008b; Kita and Kita, 2011). Synapses from GPe neurons were located proximally, close to the soma. Synapses of GPe-STN afferents had a fast GABA_A_ and a slower GABA_B_ component. Rise and decay time constants for the GABA_A_ conductance were based on Fan et al. (2012). Short-term plasticity parameters were chosen so that synapses exhibited short-term depression, as shown in Atherton et al. (2013). Parameters for the GABA_B_ synapse were taken from the model by Destexhe and Sejnowski (1995), and the decay time constant K4 was adapted so that the GABA_B_ conductance exhibited depression upon continued pre-synaptic stimulation.

**Table 3:** STN model synaptic current equations.

**Current**	**Equation**	**Location**	**Parameters**	
*I*_CTX−STN, AMPA_	${\bar{g}}_{syn}s\left(v-E_{AMPA}\right)$	distal: *x* ≥ 100μ*m*	τ_*rise*_ = 1	τ_*rec*_ = 200
			τ_*decay*_ = 4	τ_*facil*_ = 1
			*t*_*d*_ = 5.9	*U*_1_ = 0.2
			${\bar{g}}_{syn}=4.44\text{e}-3$	
*I*_CTX−STN, NMDA1_	${\bar{g}}_{syn}ms\left(v-E_{NMDA}\right)$	distal: *x* ≥ 100μ*m*	τ_*rise*_ = 3.7	τ_*rec*_ = 200
			τ_*decay*_ = 212	τ_*facil*_ = 1
			*t*_*d*_ = 5.9	*U*_1_ = 0.2
			${\bar{g}}_{syn}=5.04\text{e}-3$	
*I*_CTX−STN, NMDA2_	${\bar{g}}_{syn}ms\left(v-E_{NMDA}\right)$	proximal: *x* < 120μ*m*	τ_*rise*_ = 3.7	τ_*rec*_ = 200
			τ_*decay*_ = 80	τ_*facil*_ = 1
			*t*_*d*_ = 5.9	*U*_1_ = 0.2
			${\bar{g}}_{syn}=5.04\text{e}-3$	
*I*_GPE−STN, GABA_A__	${\bar{g}}_{syn}s\left(v-E_{GABA_{A}}\right)$	proximal: *x* < 120μ*m*	τ_*rise*_ = 2	τ_*rec*_ = 400
			τ_*decay*_ = 7	τ_*facil*_ = 1
			*t*_*d*_ = 2.0	*U*_1_ = 0.2
			${\bar{g}}_{syn}=18\text{e}-3$	
*I*_GPE−STN, GABA_B__	${\bar{g}}_{syn}\frac{G^{n}}{G^{n}+K_{d}^{n}}\left(v-E_{GABA_{B}}\right)$	proximal: *x* < 120μ*m*	τ_*rise*_ = 5	τ_*rec*_ = 400
			τ_*decay*_ = 25	τ_*facil*_ = 1
			*t*_*d*_ = 2.0	*U*_1_ = 0.2
			${\bar{g}}_{syn}=3.75\text{e}-3$	*K*_3_ = 0.098
			*n* = 4	*K*_4_ = 6.25e−3
				*K*_*d*_ = 1.4

### GPe Cell Model

GPe neurons were modeled using the baseline rat GPe neuron model by Gunay et al. (2008) (ModelDB accession number 114639). The model is based on a reconstructed morphology from the adult rat and contains nine types of ion channels with varying densities in the soma, dendrite, and axon initial segment:

(19) $$\begin{array}{l} I_{ion,j}=I_{NaF}+I_{NaP}+I_{Kv2}+I_{Kv3}\\ \;+I_{Kv4,f}+I_{Kv4,s}+I_{KCNQ}+I_{sKCa}\\ \;+I_{CaHVA}+I_{HCN,f}+I_{HCN,s}+I_{L} \end{array}$$

where *I*_*NaF*_ and *I*_*NaP*_ are the transient fast-acting and persistent sodium current, *I*_*Kv*2_ and *I*_*Kv*3_ the slow and fast delayed rectifier potassium current, *I*_*Kv*4*f*_ and *I*_*Kv*4*s*_ the fast and slow component of the A-type, transient potassium current, *I*_*KCNQ*_ the M-type potassium current, *I*_*sKCa*_ the calcium-dependent potassium current, *I*_*CaHVA*_ the high-threshold, non-inactivating calcium current (reflecting a mixture of L, N, and P/Q-type calcium channel types), and *I*_*HCN, f*_ and *I*_*HCN, s*_ the fast and slow component of the HCN channel. The equations governing the dynamics of the gating variables are listed in Table 4. The channel density distributions are those described in Gunay et al. (2008) for model *t9842*. As a source of noise, a current that with a Gaussian amplitude distribution, mean zero and standard deviation 0.0075 was added to the somatic compartment, to represent membrane voltage noise of similar amplitude the STN cell model, given the lower somatic input resistance of the STN model.

**Table 4:** GPe model intrinsic current equations from Gunay et al. (2008).

**Current**	**Equation**	**Gate**	***m*_0_**	**θ_*m∞*_**	**σ_*m∞*_**	**τ_0_**	**τ_1_**	**θ_*mτ*_**	**σ_*m*0_**	**σ_*m*1_**	**Additional parameters**
*I*_*NaF*_	${\bar{g}}_{NaF}m^{3}hs\left(v-E_{Na}\right)$	m	0	-39	5	0.028	0.028	N/A	N/A	N/A	${\bar{g}}_{NaF}=0.035$ (soma)
		h	0	-48	-2.8	0.025	4	-43	10	-5	${\bar{g}}_{NaF}=0.035$ (dendrite)
		s	0.15	-40	-5.4	10	1000	-40	18.3	-10	${\bar{g}}_{NaF}=0.5$ (axon)
*I*_*NaP*_	${\bar{g}}_{NaP}m^{3}hs\left(v-E_{Na}\right)$	m	0	-57.7	5.7	0.03	0.146	-42.6	14.4	-14.4	${\bar{g}}_{NaP}=10.15\text{e}-3$ (soma)
		h	0.154	-57	-4	10	17	-34	26	-31.9	${\bar{g}}_{NaP}=10.15\text{e}-3$ (dendrite)
		s	0	-10	-4.9	N/A	N/A	N/A	N/A	N/A	${\bar{g}}_{NaF}=4\text{e}-3$ (axon)
*I*_*Kv*2_	${\bar{g}}_{Kv2}m^{4}h\left(v-E_{K}\right)$	m	0	-33.2	9.1	0.1	30	-33.2	21.7	-13.9	${\bar{g}}_{Kv2}=0.1\text{e}-3$ (soma, dendrite)
		h	0.2	-20	-10	3400	3400	N/A	N/A	N/A	${\bar{g}}_{Kv2}=64\text{e}-3$ (axon)
*I*_*Kv*3_	${\bar{g}}_{Kv3}m^{4}h\left(v-E_{K}\right)$	m	0	-26	7.8	0.1	14	-26	13	-12	${\bar{g}}_{Kv3}=1\text{e}-3$ (soma, dendrite)
		h	0.6	-20	-10	7	33	0	10	-10	${\bar{g}}_{Kv3}=128\text{e}-3$ (axon)
*I*_*Kv*4, *f*_	${\bar{g}}_{Kv4,f}m^{4}h\left(v-E_{K}\right)$	m	0	-49	12.5	0.25	7	-49	29	-29	${\bar{g}}_{Kv4,f}=2\text{e}-3$ (soma)
		h	0	-83	-10	7	21	-83	10	-10	${\bar{g}}_{Kv4,f}=4\text{e}-3$ (dendrite)
											${\bar{g}}_{Kv4,f}=160\text{e}-3$ (axon)
*I*_*Kv*4, *s*_	${\bar{g}}_{Kv4,s}m^{4}h\left(v-E_{K}\right)$	m	0	-49	12.5	0.25	7	-49	29	-29	${\bar{g}}_{Kv4,s}=3\text{e}-3$ (soma)
		h	0	-83	-10	50	121	-83	10	-10	${\bar{g}}_{Kv4,s}=6\text{e}-3$ (dendrite)
											${\bar{g}}_{Kv4,s}=240\text{e}-3$ (axon)
*I*_KCNQ_	${\bar{g}}_{\text{KCNQ}}m^{4}h\left(v-E_{K}\right)$	m	0	-61	19.5	6.7	100	-61	35	-25	${\bar{g}}_{\text{KCNQ}}=20\text{e}-5$ (soma, dendrite)
											${\bar{g}}_{\text{KCNQ}}=4\text{e}-5$ (axon)
*I*_CaHVA_	${\bar{g}}_{\text{CaHVA}}m\left(v-E_{Ca}\right)$	m	0	-20	7	0.2	0.2	-20	N/A	N/A	${\bar{g}}_{\text{CaHVA}}=3\text{e}-5$ (soma, thick dendrites)
											${\bar{g}}_{\text{CaHVA}}=4.5\text{e}-5$ (medium dendrites)
											${\bar{g}}_{\text{CaHVA}}=9\text{e}-5$ (thin dendrites)
											${\left[{Ca}^{2+}\right]}_{i0}=5\text{e}-5$
											τ_Ca_ = 1
*I*_HCN, f_	${\bar{g}}_{\text{HCN},\text{f}}m\left(v-E_{h}\right)$	m	0	-76.4	-3.3	0	3625	-76.4	6.56	-7.48	${\bar{g}}_{\text{HCN},\text{f}}=1\text{e}-4$ (soma, dendrite)
*I*_HCN, s_	${\bar{g}}_{\text{HCN},\text{s}}m\left(v-E_{h}\right)$	m	0	-87.5	-4	0	6300	-87.5	8.9	-8.2	${\bar{g}}_{\text{HCN},\text{f}}=2.5\text{e}-4$ (soma, dendrite)

GPe neurons each had 10 excitatory afferents from STN neurons, 6 inhibitory afferents from GPe-GPe collaterals, and 30 inhibitory afferents from iMSN (Table 5). The location of synapses on GPe neurons and axonal propagation delays were based on experimental observations reported in the literature (Table 1). Relative magnitudes of synaptic conductances were chosen to bring the population firing rate into the reported range for the rat (Mallet et al., 2008b; Kita and Kita, 2011). Rise and decay time constants for AMPA conductances were set to 1 and 4 ms, respectively, and for GABA_A_ conductances they were set to 2 and 5 ms, respectively. Synapses from STN neurons were located distally, in the dendritic tree. They consisted of an AMPA component and were modeled using Tsodyks-Markram dynamics. The parameters describing short-term plasticity dynamics were chosen to match traces reported in Hanson and Jaeger (2002). Synapses from GPe were located proximally, near the soma and had both a fast GABA_A_ component with Tsodyks-Markram dynamics, and a slow metabotropic GABA_B_ component. Short-term plasticity parameters were chosen so that synapses exhibited short-term depression (Miguelez et al., 2012). Synapses from striatal neurons had a GABA_A_ component and were made to exhibit short-term facilitation based on Miguelez et al. (2012).

**Table 5:** GPe model synaptic current equations.

**Current**	**Equation**	**Location**	**Parameters**	
*I*_STN−GPE, AMPA_	${\bar{g}}_{syn}s\left(v-E_{AMPA}\right)$	distal: *x* ≥ 100μ*m*	τ_*rise*_ = 1	τ_*rec*_ = 200
			τ_*decay*_ = 4	τ_*facil*_ = 800
			*t*_*d*_ = 2	*U*_1_ = 0.1
			${\bar{g}}_{syn}=3.75\text{e}-4$	
*I*_GPE−GPE, GABA_A__	${\bar{g}}_{syn}s\left(v-E_{GABA_{A}}\right)$	proximal: *x* < 200μ*m*	τ_*rise*_ = 2	τ_*rec*_ = 400
			τ_*decay*_ = 5	τ_*facil*_ = 1
			*t*_*d*_ = 0.5	*U*_1_ = 0.2
			${\bar{g}}_{syn}=2\text{e}-4$	
*I*_GPE−GPE, GABA_B__	${\bar{g}}_{syn}\frac{G^{n}}{G^{n}+K_{d}^{n}}\left(v-E_{GABA_{B}}\right)$	proximal: *x* < 200μ*m*	τ_*rise*_ = 5	*K*_3_ = 0.098
			τ_*decay*_ = 25	*K*_4_ = 6.25e−3
			*t*_*d*_ = 0.5	*K*_*d*_ = 1.4
			${\bar{g}}_{syn}=0.4\text{e}-4$	*n* = 4
*I*_iMSN−GPE, GABA_A__	${\bar{g}}_{syn}s\left(v-E_{GABA_{A}}\right)$	proximal: *x* < 200μ*m*	τ_*rise*_ = 2	τ_*rec*_ = 1
			τ_*decay*_ = 5	τ_*facil*_ = 200
			*t*_*d*_ = 5	*U*_1_ = 0.3
			${\bar{g}}_{syn}=3\text{e}-4$	

### Modeling the Parkinsonian State

To model the parkinsonian state, the biophysical properties of the network and cell models were modified based on experimental observations made in the dopamine depleted and control conditions as reported in the literature. Various biophysical parameters, including synaptic strengths and time constants are affected by dopamine depletion, and were adjusted as detailed below. Scaling factors for synaptic and ionic conductances were set to experimentally reported values where available. Otherwise they were chosen to bring the mean population firing rates into physiological ranges reported for the rat in a state of cortical activation during light anesthesia (Mallet et al., 2008b; Kita and Kita, 2011).

The mean firing rate of STN surrogate spike sources was increased from 14.6 to 29.5 Hz in the parkinsonian state (Mallet et al., 2008b). The peak GABA_A_ and GABA_B_ conductance of GPe to STN synapses was increased by 50% and the GABA_B_ decay time constant increased by 2 ms to model the increase in the number of contacts, vesicle release probability, and decay kinetics of GPe afferents (Fan et al., 2012). To model the reduction in cortico-STN axon terminals and their dendritic targets (Chu et al., 2017; Wang et al., 2018) the number of CTX-STN afferents was reduced to 70% of the normal condition, corresponding to the ratio of vGluT1 expression in the normal and dopamine depleted condition used to label axon terminals (Chu et al., 2017). To model functional strengthening of remaining synapses, the AMPA and NMDA peak conductances of remaining synapses were multiplied by the ratio of the current scaling factors reported in Shen and Johnson (2005) to the fraction of remaining synapses. The effect of functional strengthening and weakening of the CTX-STN projection was further investigated by systematically varying the peak synaptic conductances in the simulations experiments. Finally, HCN currents were reduced by 50% to model reduced depolarization and spontaneous activity after dopamine depletion (Zhu et al., 2002; Cragg et al., 2004) and modulation of HCN current by D2R receptors (Yang et al., 2016).

In GPe neurons the peak AMPA conductance of STN afferents was increased by 50% to model the modulatory effect of dopamine on glutamergic excitatory currents (Johnson and Napier, 1997; Hernández et al., 2006; Kita, 2007). The strengthening of GPe-GPe collaterals (Miguelez et al., 2012; Nevado-Holgado et al., 2014) was modeled by increasing the peak GABA_A_ and GABA_B_ conductances by 50%. The mean firing rate of GPe surrogate spike sources was decreased from 33.7 to 14.6 (Mallet et al., 2008a). Finally, the HCN channel conductance was decreased by 50% in accordance with experimental data (Chan et al., 2011).

In simulations without oscillatory inputs, cortical projection neurons were modeled as Poisson spike generators firing at 10 Hz, a multiple of the experimentally reported rate of 2.5 Hz (Li et al., 2012), so that each synapse represented the combined inputs of four pre-synaptic neurons (making use of the additive property of the Poisson distribution). In simulations with oscillatory inputs, oscillatory spike trains were generated as follows: on top of the aforementioned background firing pattern, bursts were added in each period of a regular oscillation at the chosen oscillation frequency. In each period of the oscillation 10% of neurons were selected randomly to emit a burst. The onset time of the burst was the same in each selected neuron, so that bursts occurred in-phase between neurons, but the number of spikes in a burst was variable with inter-spike intervals sampled from the interval [5, 6] ms. All background spikes occurring in a time window centered on a burst were deleted to prevent unrealistically high inter-spike intervals. **Figure 7D** shows a rastergram with representative spike trains generated using this method.

The increase in excitability and spontaneous activity of iMSN (Kita and Kita, 2011; Fieblinger et al., 2014) was modeled by increasing the mean firing rate of the Poisson spike generators from 1.5 to 6.64 Hz. In experiments where iMSN cells fired oscillatory bursts the same algorithm as described for cortical projection neurons was used. The modulation of GABAergic transmission from iMSN to GPe neurons (Cooper and Stanford, 2001; Shin et al., 2003) was modeled by increasing the initial release probability and the peak GABA_A_ conductance of synapses by 50%.

### Simulation Details

The model was simulated in the NEURON simulation environment (Hines and Carnevale, 1997) and implemented in Python. The default fixed time step integrator with a time step of 0.025 ms was used for all simulations. Compartmental membrane voltages were initialized to a random value between −63 and −73 mV in GPe and between −60 and −70 mV in STN cells. Gating variables were initialized to their equilibrium values for the initial membrane voltage. Simulation data for the first 2,000 ms of each simulation were discarded, and the analyzed intervals were of duration 4,000 ms unless otherwise noted. Simulations were run on the UCD Sonic cluster using 8 parallel processes per simulation on a single computing node, consisting of two Intel Ivybridge E5-2660 v2 CPUs (10 cores per CPU).

### Signal Analysis

Signal analyses were performed using the SciPy toolbox (Jones et al., 2001) for Python. Power spectral densities (PSDs) were calculated using Welch's periodogram method, using overlapping segments of 2 s duration with 50% overlap and a Hanning window. Given the sampling period of 0.05 ms this led to a frequency resolution of 0.5 Hz. The population PSD was calculated as the mean PSD of all somatic membrane voltages. The instantaneous phase of each population was estimated by applying the Hilbert transform to the average somatic membrane voltage of cells in the population, after band-pass filtering using a neutral-phase filter (Butterworth filter, 4th order, command *sosfiltfilt*) in an 8 Hz wide frequency band centered on the dominant oscillation frequency. For populations that were modeled as surrogate spike trains (cortex and striatum), artificial membrane voltage signals were first constructed by convolving the spike trains with a typical action potential waveform. Bursts were detected using a simple algorithm where a burst consisted of a minimum of four spikes with inter-spike intervals (ISIs) ≤ 20 ms.

## Results

The STN-GPe pacemaker hypothesis was first investigated by modeling cortical inputs to the STN as Poisson spike generators without any periodic or oscillatory component. Cortical patterning of neural activity in the STN-GPe network via the hyperdirect pathway was then investigated by modeling cortical input to the STN inputs as periodically bursting spike trains. To investigate whether changing the excitation-inhibition balance in STN and GPe contributed to changes in spontaneous synchronization and functional coupling between nuclei, the ratio of excitation and inhibition was systematically increased by altering the strength of individual projections between nuclei. The ratio of total excitatory to inhibitory synaptic currents (E/I ratio) was altered by scaling the peak conductance of all synapses belonging to a given projection known to be strengthened or weakened by dopamine depletion. The role of additional oscillatory inputs entering the STN-GPe network via the indirect striato-pallidal pathway and their phase relationship to cortical inputs were then investigated.

### The Balance of Excitation and Inhibition Balance in the STN Affects the Oscillation Frequency of the STN-GPe Network and Firing Mode of STN Neurons

**Figure 2:**
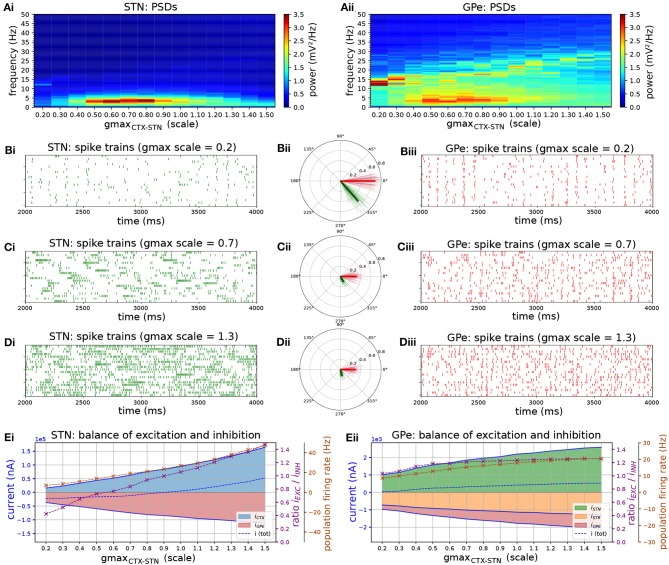
The level of excitation by cortex determines firing patterns and oscillation frequency in the autonomous STN-GPe network. Behavior of the autonomous STN-GPe network for increasing values of the CTX to STN synaptic conductance. **(A)** Mean PSD of the somatic membrane voltages of STN **(Ai)** and GPe **(Aii)** neurons. **(B–D)** Representative spike trains and phase vectors for STN (column i, green) and GPe population (column iii, red) for three different scale factors of the CTX to STN conductance [scale 0.2; 0.7; 1.3 in rows **(C–E)**, respectively]. Column ii shows phase vectors of the STN and GPe populations (in green; red, respectively, mean population vectors plotted as thick solid lines and cell vectors as thin transparent lines) reflecting phase locking to the instantaneous GPe phase. Phase vectors were measured with respect to the instantaneous phase of the GPe population extracted using a bandpass filter with passband of 8 Hz centered on the frequency bin with maximum power in the 13–30 Hz band. **(E)** Balance of excitation and inhibition in the STN **(Ei)** and GPe **(Eii)** based on synaptic currents recorded in three neurons. Population firing rate (brown), E/I ratio (purple), and net synaptic current (blue). Shaded areas represent estimated total synaptic current from one pre-synaptic population during a simulation. Total current was estimated by recording all synapses on 3 randomly selected cells in each population and adjusting for the true number of cells.

Increasing the strength of the CTX-STN projection by increasing the conductance of cortico-subthalamic synapses revealed parameter regimes that favored low frequency bursting in STN neurons and phase-locking to an emergent beta-band rhythm in the STN-GPe network (Figures 2A–D). For lower values of synaptic conductance the network exhibited synchronous oscillatory activity at 12–13 Hz (Figure 2A), with both STN and GPe neurons entrained to the oscillation (Figures 2Bi–iii). This high entrainment regimen coincided with low neuronal firing rates (Figure 2E) where short spike sequences, mostly singlets and doublets, showed a high phase preference as evidenced by the high population and individual neuronal phase vector lengths (Figure 2Bii).

Increasing the synaptic conductance caused a proportional increase in excitatory current to the STN (Figure 2E, blue area), with a corresponding increase in inhibition (red area) as a result of the negative feedback structure of the STN-GPe loop. However, because the GPe population exhibited a saturating population firing rate curve (Figure 2Eii), feedback inhibition to STN was outpaced by cortical excitation, resulting in a shift to net excitation (E/I > 1). This saturating firing rate curve in the GPe was a result of two negative feedback mechanisms that have a homeostatic effect on the GPE's E/I ratio: reciprocal inhibition through intra-GPe colaterals and short-term depression of STN to GPe synapses (Hanson and Jaeger, 2002). The increase of excitatory drive in the network increased the frequency at which oscillations emerged within the network (Figure 2Aii, peak in the PSD is shifted), though the level of synchronization of neurons was relatively weak. This was particularly the case in the STN, as evidenced by the low phase vector lengths (Figures 2Cii,Dii,Eii). Despite the lower vector lengths, reflecting more dispersed spike timings within a period of the oscillation, spikes in both STN and GPe neurons showed a consistent phase preference with respect to the ongoing oscillation, as evidenced by the alignment of individual neuronal and population phase vector. The STN population vector led that of GPe by 45 degrees indicating that STN neurons excited GPe neurons which responded with a delay of 10 ms, resulting in a wave of inhibition to the STN with a long recovery period comparable to the oscillation period. Although excitation outpaced inhibition in STN neurons, higher inhibitory currents resulted in increased transient inhibition of STN dendrites, engaging the ion channels underlying burst responses. This brought STN neurons into a slow burst firing mode characterized by sparse, strong bursts (Figure 2Ci). These low-frequency fluctuations in firing rate were transmitted to GPe neurons as evident in the power spectra of both nuclei (Figures 2Ai,ii).

**Figure 3:**
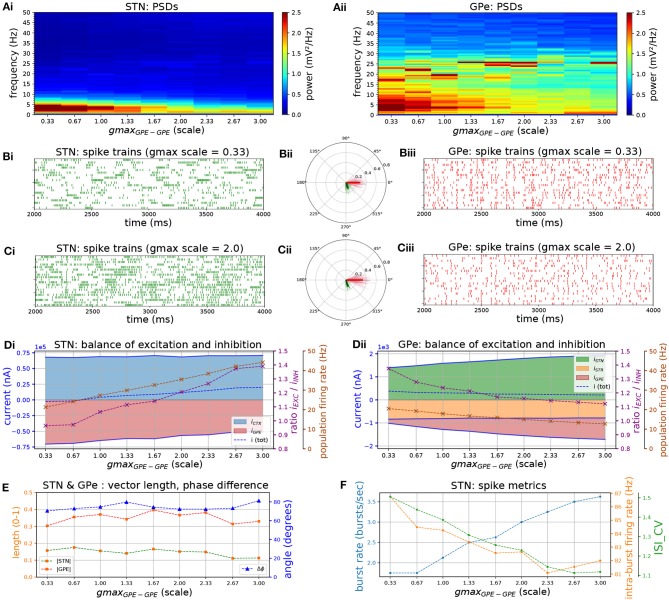
Increasing the level of collateral GPe-GPe inhibition shifts the excitation-inhibition balance in STN and GPe in opposite directions. Behavior of the autonomous STN-GPe network for increasing values of GPe-GPe synaptic conductance. **(A)** Mean PSD of the somatic membrane voltages of STN **(Ai)** and GPe **(Aii)** neurons. **(B,C)** Representative spike trains and phase vectors for STN (column i, green) and GPe population (column iii, red) for two values of the GPe to GPe conductance [scale 0.33; 2.0 in rows **(B,C)**, respectively]. Column ii shows phase vectors of the STN and GPe populations (in green; red, respectively, mean population vectors plotted as thick solid lines and cell vectors as thin transparent lines) reflecting phase locking to the instantaneous GPe phase. **(D)**: Balance of excitation and inhibition in the STN **(Di)** and GPe **(Dii)** based on synaptic currents recorded in three neurons. Mean population firing rate (brown), E/I ratio (purple), and net synaptic current (blue). Shaded areas represent estimated total synaptic current from one pre-synaptic population during a simulation. **(E)** Population vector length and angle of STN and GPe population (green; red, respectively). **(F)** Metrics that characterize bursting in STN neurons: median burst rate, intra-burst firing rate, and coefficient of variation of ISIs across all STN cells.

Increasing the strength of GPe-GPe colaterals (Figure 3) similarly increased the level of excitation of STN neurons but by a different mechanism. By increasing self-inhibition within the GPe, and thereby decreasing inhibition of targets in the STN (Figure 3D, red area), the E/I ratio in both populations moved in opposite directions. As the E/I ratio in STN increased toward dominant excitation (Figure 3D), neural activity shifted from strong low-frequency bursting (characterized by a high intra-burst firing rate and high low-frequency power) (Figures 3Ai,Bi,F) toward more regular firing with decreasing coefficient of variation of inter-spike intervals (CV_ISI_) and intra-burst firing rate (Figures 3Ci,F). The E/I ratio and population firing rate in the GPe showed a saturating characteristic (Figure 3Dii) caused by the negative feedback structures inherent in the loop as before (Figure 3D) as it was progressively disinhibited. GPe neurons were more strongly entrained to the emergent oscillation (17–26 Hz) whereas STN spiking showed a weaker phase preference (Figures 3Bii,Cii,E). This result was the same whether the instantaneous phase was extracted from the STN or GPe population.

### Strength and Time-Course of GPe-STN Inhibition Controls Bursting and Phase-Locking in STN Neurons

Following dopamine depletion the inhibitory GPe-STN connection is strengthened by a proliferation of synapses and increased decay kinetics of GABA currents (Fan et al., 2012). Moreover, the expression of both GABA_A_ (Fan et al., 2012) and GABA_B_ (Shen and Johnson, 2005) receptors is upregulated leading to larger evoked synaptic currents. To investigate the effects of increased inhibition and altered kinetics of inhibitory post-synaptic currents (IPSC) in STN neurons on network activity patterns, an increase in the GABA_A_ and GABA_B_ conductances was simulated and the relative contribution of both currents was altered.

**Figure 4:**
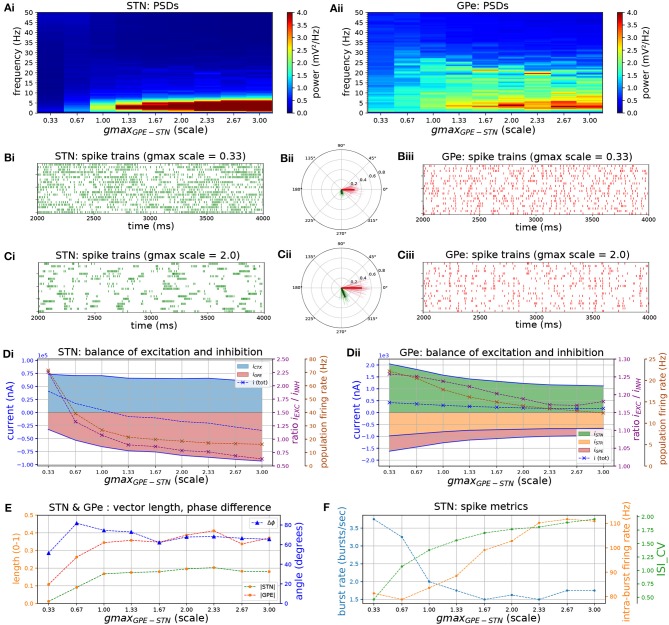
Increasing the level of GPe-STN inhibition shifts STN to a low-frequency burst firing mode. Behavior of the autonomous STN-GPe network for increasing values of the GPe to STN synaptic conductance. **(A)** Mean PSD of the somatic membrane voltages of STN **(Ai)** and GPe **(Aii)** neurons. **(B,C)** Representative spike trains and phase vectors for STN (column i, green) and GPe population (column iii, red) for two values of the GPe to STN conductance [scale 0.33; 2.0 in rows **(C,D)**, respectively]. Column ii shows phase vectors of the STN and GPe populations (in green; red, respectively, mean population vectors plotted as thick solid lines and cell vectors as thin transparent lines) reflecting phase locking to the instantaneous GPe phase. **(D)** Balance of excitation and inhibition in the STN **(Di)** and GPe **(Dii)** based on synaptic currents recorded in three neurons. Population firing rate (brown), E/I ratio (purple), and net synaptic current (blue). Shaded areas represent estimated total synaptic current from one pre-synaptic population during a simulation. **(E)** Population vector length and angle of STN (green) and GPe (red) population. **(F)** Metrics that characterize bursting in STN neurons: median burst rate, intra-burst firing rate, and coefficient of variation of ISIs across all STN cells.

Increasing the conductance of both GABA_A_ and GABA_B_ synapses lead to an increase in low-frequency bursting of STN neurons (Figures 4A–C). Bursting was periodic at low frequencies (~ 2–5 Hz) but was not synchronized between cells (Figure 4Ci). Increasing the conductance also shifted the firing mode of STN neurons toward longer bursts with higher intra-burst firing rate against a lower background firing rate, characterized by a high coefficient of variation of ISIs (Figures 4D,F). Bursting with high intra-burst firing rates is mediated by a shift toward net inhibition in STN neurons (Figure 4D), leading to increased availability of voltage-sensitive Na^+^ and Ca^2+^ channels through de-inactivation at hyperpolarized membrane voltages (Baufreton et al., 2005; Gillies and Willshaw, 2005; Hallworth and Bevan, 2005). The GPe neuron model does not possess the same high density of Ca^2+^ channels that underlies plateau potentials and strong bursting, and therefore has a lower tendency toward burst firing. While STN neurons were more weakly entrained to the beta oscillation they preferentially fired in an interval leading the GPe by 65 degrees (Figures 4B,C,E). The shift toward low-frequency, fast bursting coincided with an increase in synchronization in the network, as measured by the population vector length of the STN and GPe.

**Figure 5:**
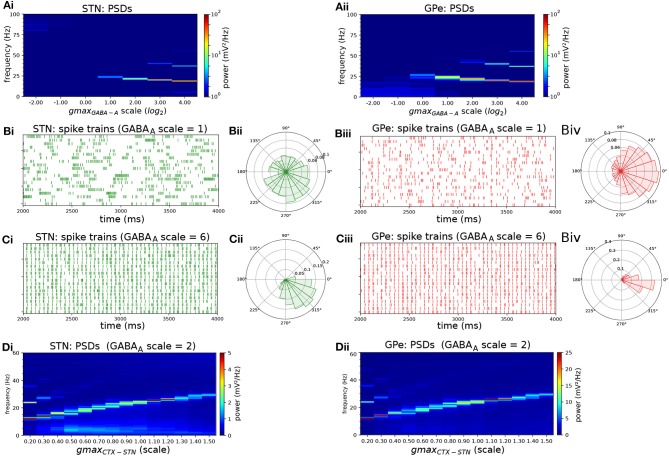
Endogenous oscillations in the STN-GPe network are strengthened by shifting the GPe-STN synaptic current from slow GABA_B_ receptors to fast GABA_A_ receptors. Behavior or the STN-GPe network for increasing values of the GABA_A_ to GABA_B_ conductance ratio. The GABA_B_ conductance of the GPe to STN projection was decreased by 50% and the GABA_A_ conductance was increased progressively. **(A)** Mean PSD of the somatic membrane voltages of STN **(Ai)** and GPe **(Aii)** neurons. **(B,C)** Representative spike trains and phase histograms of STN (green) and GPe neurons (red) in baseline model without scaling of conductances **(B)** and model where GABA_A_ conductance was scaled by a factor 6 and GABA_B_ conductance was scaled by factor 0.2, chosen so that the E/I ratio was close to that in the baseline model (**B**: baseline model, E/I ratio was 0.89; 0.97 in STN, GPe respectively; **C**: scaled conductances, ratio was 0.89; 0.93). The presence of stronger GABA_B_ currents results in higher phase dispersion **(B)** compared to the case with weaker GABA_B_ currents and stronger, fast GABA_A_ currents **(C)**. **(D)** Mean PSD of somatic membrane voltages of STN **(Di)** and GPe **(Dii)** neurons for increasing CTX-STN conductance and adjusted GABA_A_ to GABA_B_ ratio. The GABA_B_ conductance of GPe to STN synapses was halved, and the GABA_A_ conductance was doubled.

To investigate the effect of IPSC kinetics on the generation of beta oscillations within the network, the relative strength of the GABA_A_ and GABA_B_-mediated current was changed by decreasing the GABA_B_ conductance by 50% and increasing the GABA_A_ conductance progressively (Figure 5). As this increased the level of inhibition in STN neurons, it resulted in a small shift in the oscillation frequency across the parameter sweep (Figure 5A). The simulations results showed that the slow nature of the GABA_B_-mediated current prevented GPe neurons from patterning their targets with short duration IPSC required for strong entrainment in the 20–30 Hz range. When the GABA_A_ conductance was increased, and the GABA_B_ conductance decreased accordingly, both STN and GPe neurons entrained strongly to the beta rhythm as evident in phase histograms and spike trains (Figures 5B,C). When the experiment of Figure 2 was repeated in the adjusted network with a higher GABA_A_ to GABA_B_ ratio, the oscillation frequency in both STN and GPe also showed a clear sensitivity to the strength of the Poisson distributed cortical excitatory input (Figure 5D).

### STN-GPe Network Shows Resonant Properties and Phase Locks to Cortical Beta Inputs

**Figure 6:**
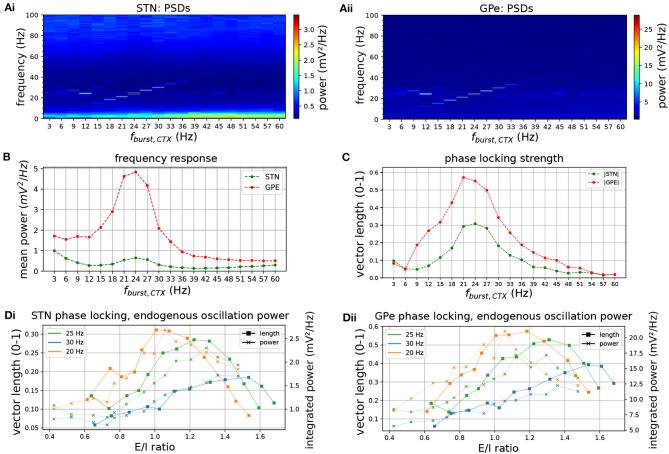
Frequency response and phase locking of the STN-GPe network to cortical oscillatory bursting inputs. Sweep of cortical oscillation frequency (top row) and phase locking to cortical oscillations for increasing CTX-STN synapse strength (bottom row). **(A)** Mean PSD of somatic membrane voltages in STN **(Ai)** and GPe **(Aii)** for increasing oscillatory bursting frequency. **(B)** Mean PSD of the somatic membrane voltages of STN (green) and GPe (red) neurons, averaged within a 5 Hz wide frequency band centered on the cortical oscillation frequency. **(C)** Population vector length, indicating strength of phase locking to the cortical oscillation of STN (green) and GPe (red) neurons. **(D)** Change in population vector length (solid lines) for a fixed cortical oscillation frequency (20 Hz, 25 hz, 30 Hz in green, blue, orange, respectively) and increasing CTX-STN input synaptic conductance, reflected in an increased ratio of excitation to inhibition (E/I ratio). Endogenous oscillation power in simulations without oscillatory cortical input is plotted for comparison (dotted lines, power integrated in 5 Hz band centered on cortical frequency in equivalent simulation with cortical inputs). An increased E/I ratio results in maximum phase locking at a higher oscillation frequency, and power of endogenous oscillations follows trend of phase locking strength.

**Figure 7:**
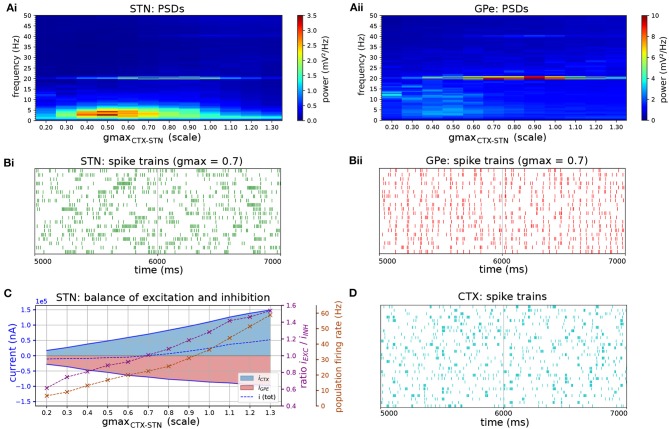
Response of STN-GPe network to cortical oscillatory bursting at 20 Hz. **(A)** Mean PSD of somatic membrane voltages in STN **(Ai)** and GPe **(Aii)** as a function of the synaptic conductance (scale factor) of CTX-STN inputs. The peak at 20 Hz reaches a maximum when synapses are at 70% of their baseline strength, whereas the peak in low-frequency power (2-5 Hz) occurs at 50%. **(B)** Representative spike trains of STN (**Bi**, green) and GPe neurons (**Bii**, red) in simulation with synaptic conductances scaled to 70%, corresponding to maximum phase locking and 20 Hz power. **(C)** Balance of excitation and inhibition in the STN **(Ci)** and GPe **(Cii)** based on synaptic currents recorded in three neurons. Population firing rate (brown), E/I ratio (purple), and net synaptic current (blue). **(D)** Cortical oscillatory bursting pattern illustrated using representative spike trains. In each cycle of the oscillation 10% of cells were selected at random to fire a burst in phase with the oscillation, with a variation of 1 ms on the onset and spike timings.

The degree of phase locking of the STN-GPe network to synchronous cortical rhythms and its sensitivity to intrinsic network parameters was then examined. The network was simulated with cortical inputs modeled as spike trains exhibiting sparse, synchronous bursts. The frequency of the synchronous cortical inputs was first increased from 3 to 60 Hz and the frequency response and phase locking strength of the STN-GPe loop was estimated (Figures 6A–C). Spectral power and phase locking, measured by the population vector length, were strongest when the cortical oscillation frequency was close to the network's endogenous oscillation frequency (Figures 6B,C), indicating a resonance effect. Spectral power at the oscillation frequency was increased considerably above that observed for Poisson distributed cortical inputs (compare Figures 6Ai,ii to Figures 2Ai,ii). Moreover, the frequencies that were amplified by the STN-GPe network corresponded well to the beta-band, i.e., 13–30 Hz (Figure 2B). To study the dependence of the resonance peak on the excitation-inhibition balance in the STN, the cortical input strength was then varied while the oscillation frequency remained fixed (Figure 6D). The range of synaptic conductances was chosen so that the STN population firing rate traversed the experimentally reported range of 17–37 Hz (Mallet et al., 2008b; Kita and Kita, 2011) in the dopamine depleted state during cortical activation (Figure 7C). Maximum phase locking coincided with frequency of maximum endogenous oscillation power observed in the absence of oscillatory inputs (Figures 2Di,ii). The results demonstrate how the resonant frequency of the network can be shifted by changing the excitation-inhibition balance, biasing the network toward a slower or faster oscillation. GPe neurons synchronized stronger to the oscillatory input compared to STN neurons (Figures 6B,C, 7Bi,ii), which showed a tendency to burst, mirroring the results for spontaneous synchronization in the autonomous STN-GPe network. Analogous to the autonomous loop, when the slow bursting behavior was reduced by shifting the GPe to STN synaptic current from GABA_B_ to faster GABA_A_ receptors, synchronization and phase locking of both STN and GPe neurons was greatly increased.

### Influence of Phase Relationship Between Cortical and Striatal Beta Inputs

Striatal microcircuits exhibit beta-band oscillations in healthy primates (Feingold et al., 2015) and parkinsonian rodent models (McCarthy et al., 2011; Sharott et al., 2017) and have been hypothesized to be part of the pacemaking circuit that generates them. In the previous section, the STN-GPe network was shown to generate weak beta-band oscillations in the absence of exogenous beta inputs (Figures 2, 3, 4), and to phase lock to cortical beta-band inputs which amplified oscillatory activity (Figure 6). A potential role of the pallido-striatal loop could be to amplify beta-band oscillations in the STN-GPe network to a more pathological level, as part of a double resonant loop converging on the GPe. A suggested mechanism is that altered striatal activity in PD could shift the phase of firing of the GPe relative to the STN to one that supports STN phase locking through increasing the availability of Na^+^ and Ca^2+^ channels post-inhibition and pre-excitation (Baufreton et al., 2005; Mallet et al., 2008a, 2012). Alternatively, oscillations that originate in striatal circuits could be transmitted via the striato-pallidal projection and thus introduced into the STN-GPe network (McCarthy et al., 2011; Corbit et al., 2016). Of the two loops converging on GPe neurons, inhibitory striatal afferents would be better suited to interrupt ongoing activity and influence the phase compared to excitatory STN afferents. Hence, the iMSN to GPe projection could play an important role in patterning neural activity in the STN-GPe network.

Phase vector plots in the previous section show that STN and GPe neurons settle into a particular phase relationship where STN leads GPe by 60–90 degrees which contributed to sustaining beta-band oscillations. We hypothesized that inhibitory inputs from the striatum would either disrupt this phase relationship, thereby suppressing beta-band oscillations, or reinforce them depending on where in the phase of the beta oscillation they arrive. To investigate this hypothesis, surrogate striatal spike trains exhibiting beta frequency bursts were generated and the phase with respect to the incoming cortical oscillation was increased in increments of 45 degrees by varying the onset time of bursts. As iMSN-GPe synapses exhibit short-term facilitation, bursts administered through this projection led to an increase in inhibition to the GPe that was greater than the relative increase in spike rate. To compensate for this effect and maintain a physiological firing rate range of the GPe neurons, the peak conductance of iMSN-GPe synapses was reduced by 60%.

**Figure 8:**
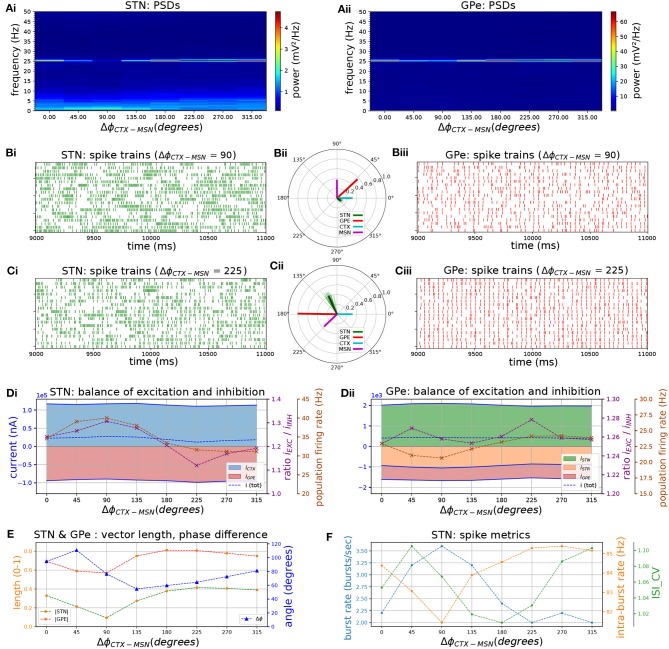
The phase relationship between cortical and striatal beta-band inputs to the STN-GPe network affects the strength of phase-locking by setting the relative timing of excitatory and inhibitory STN afferents. Response of the STN-GPe network to oscillatory bursting inputs applied via both cortico-subthalamic (CTX-STN) and striato-pallidal (iMSN-GPe) afferents. The phase difference between cortical and striatal oscillatory bursts was increased in steps of 45°. All phase vectors were measured with respect to the instantaneous phase of the cortical oscillation. **(A)** Mean PSD of the somatic membrane voltages of STN **(Ai)** and GPe **(Aii)** neurons, showing weakening and strengthening of oscillations as relative phases of inputs are rotated. **(B,C)** Representative spike trains and phase vectors of STN (column i, green) and GPe population (column iii, red) for CTX-iMSN phase difference of 90° **(C)** and 225° **(D)**. Column ii shows phase vectors of the STN, GPe, CTX, iMSN populations (in green; red; blue; purple, respectively; mean population vectors plotted as thick solid lines and cell vectors as thin transparent lines). **(D)** Balance of excitation and inhibition in the STN **(Di)** and GPe **(Dii)** based on synaptic currents recorded in three neurons. Population firing rate (brown), E/I ratio (purple), and net synaptic current (blue). Shaded areas represent estimated total synaptic current from one pre-synaptic population during a simulation. **(E)** Population vector length and angle of STN (green) and GPe (red) population. **(F)** Metrics that characterize bursting in STN neurons: median burst rate, intra-burst firing rate, and coefficient of variation of ISIs across all STN cells.

Varying the phase of striatal relative to cortical bursts revealed that populations connected by an inhibitory projection, i.e., iMSN, GPe, and STN maintained a rigid phase relationship with respect to the cortical oscillation (Figure 8: population vectors in green, red, purple formed a rigid frame that rotated relative to the cyan-colored cortical population vector). The local maximum in phase locking occurred when excitatory CTX and inhibitory GPe afferents to STN fired in anti-phase, occurring when the CTX-iMSN phase difference was set to 225 degrees (Figures 8B,D,Ei). This supports the feedback inhibition hypothesis where cortical patterning is promoted when GPe-STN inhibition is offset in phase relative to cortical excitation in PD (Baufreton et al., 2005; Mallet et al., 2008a, 2012). The changing phase relationship of cortical spiking relative to the three other populations also shifted the balance of excitatory and inhibitory currents in the STN (Figure 8Ai). Maximum phase locking occurred where the STN was maximally inhibited (E/I ratio ≈ 1.1, population firing rate ≈ 21 Hz), whereas minimum phase locking coincided with maximum excitation (E/I ratio ≈ 1.3, population firing rate ≈ 40 Hz). In the GPe this relationship between phase locking strength and firing rate was reversed (Figure 8Ai) whereas the relationship with E/I ratio showed no clear trend. The optimal phase relationship of 225 degrees further strengthened phase locking to the applied beta rhythm compared to the situation with only cortical oscillatory inputs. Maximum vector length was increased by a factor of two, confirming increased synchronization, in both populations when compared to the case where only cortical beta frequency inputs were simulated. Maximum power at the oscillation frequency was also increased by a factor of 2.7 in STN and 5.2 in GPe.

### Mechanism of Phase Locking

**Figure 9:**
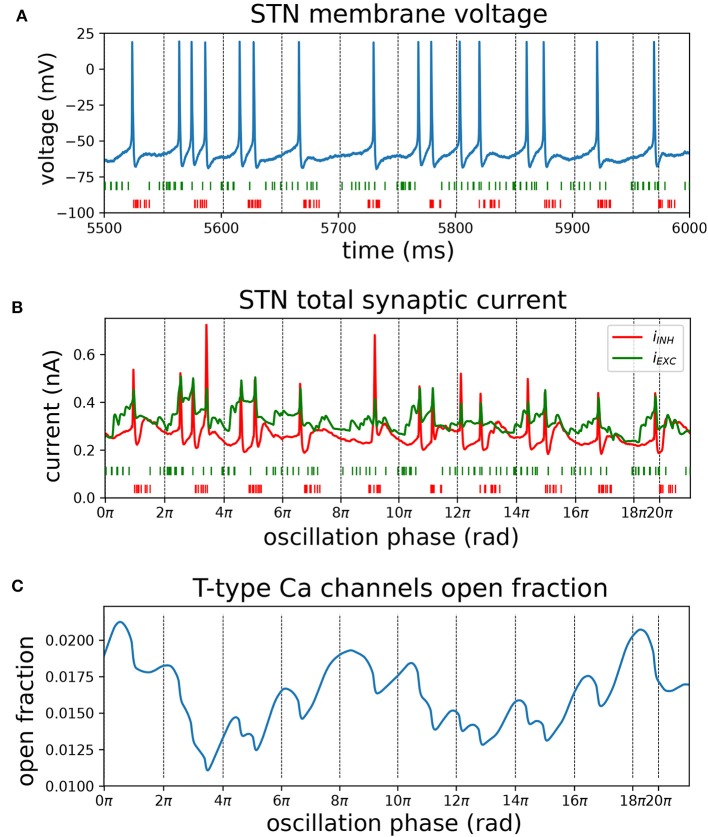
Mechanisms contributing to phase locking of STN cells to cortical beta oscillations. Recordings of synaptic currents and T-type calcium (CaT) channel inactivation from an identified phase-locked STN cell during a simulation with high phase locking (analogous to Figure 8D, cortical and striatal beta bursts at 20 Hz with phase difference of 225 degrees). Inactivation variables were recorded from each compartment with CaT ion channels and averaged over all compartments in the cell. Zero-crossings of the instantaneous beta phase are indicated using vertical dotted lines. **(A)** Somatic membrane voltage during phase-locked interval (blue). Spike trains from excitatory (green) and inhibitory (red) afferents to the cell were pooled. **(B)** Total excitatory and inhibitory synaptic current (in green; red, respectively) and pooled spike trains underneath. **(C)** Mean CaT channel inactivation across the cell's dendritic tree. High values correspond to de-inactivation. Transient de-inactivation approximately one half period after an inhibitory barrage engages depolarizing T-type *Ca*^2+^ current and contributes to phase-locked spiking.

To further illustrate the interaction between synaptically coupled STN and GPe neurons in the model under conditions of synchronous oscillatory beta-band activity, the mechanism of phase locking of STN cells is presented in Figure 9. Pooled cortical spike trains (Figures 9A,B, green) illustrate how sparse cortical beta bursts (Figure 7B) result in distributed synaptic inputs to individual STN neurons that are not tightly phase locked, but have a combined firing rate that is modulated at the beta frequency. While these exogenous cortical inputs had high spike timing variability, STN and GPe spikes became highly structured and tightly locked to the beta oscillation through the feedback inhibition mechanism. The cortical beta modulation is transmitted to the STN and then to the GPe through their excitatory projections (see phase vectors in Figure 8Dii). When the inhibitory feedback arrives back in STN this shuts down spiking (Figure 9A) and simultaneously primes the cell for the next period of increased cortical excitation by de-inactivating Ca^2+^ channels (Figure 9C) and Na^+^ channels. As the cortical firing rate rises again, synaptic currents (Figure 9B) combine with dendritic Ca^2+^ currents to overcome any lingering inhibition and cause the next wave of phase-locked STN spikes. The striatal beta inputs further decreased spiking variability of GPe neurons by narrowing their time window of firing through phasic inhibition (purple phase vector in Figure 8Dii).

## Discussion

A new model of the STN-GPe network is presented that incorporates biophysically detailed multi-compartment cell models. The individual STN and GPE cell models capture the interaction of intrinsic and synaptic membrane currents with non-uniform subcellular distributions across the dendritic structure, which can not be captured in single compartment models. The model illustrates how phase locking of STN and GPe neurons, and increased bursting of STN neurons, can arise from the interaction of these currents when their relative strengths and temporal relationships are altered. The STN-GPe model network showed an intrinsic susceptibility to beta-band synchrony that manifest as weak, autonomously-generated endogenous oscillations and selective amplification of exogenous beta-band synaptic inputs at the network's preferred oscillation frequency. The frequency at which endogenous beta oscillatory activity occurred varied with the ratio of excitatory to inhibitory currents to the STN. Varying the phase relationships between external beta-frequency inputs to the network through cortical and striatal pathways further increased or suppressed the level of amplification of cortical beta inputs by modulating the temporal dispersion of action potentials in STN neurons and thereby influencing the precision of phase locking. Varying synaptic strengths within the network affected the balance of excitation and inhibition in both STN and GPe neurons and produced a rich set of behaviors, not only modulating firing rates but also affecting synchronization and bursting properties of neurons. Homeostatic mechanisms mediated by feedback connections and short-term synaptic plasticity dynamics served to stabilize the excitation-inhibition balance in the GPe and reduced the sensitivity of its population firing rate to variations in pre-synaptic rates.

### Oscillatory Properties of the Multi-compartmental STN-GPe Network

In the autonomous STN-GPe network, under conditions of Poisson distributed external synaptic inputs, STN neurons exhibited weak synchronization to the endogenous beta rhythm but retained a weak phase preference with respect to the stronger oscillation in the GPe population (Figures 2–4). The synchronization strength of STN neurons was found to depend on the relative strength of GABA_A_ and GABA_B_ receptors in STN dendrites (Figure 5), with an increase in the proportion of fast-acting GABA_A_ receptors resulting in an increase in the strength of oscillation. The endogenous oscillation frequency of the STN-GPe network was further influenced by the balance of excitatory and inhibitory currents in the STN. This balance affected the net level of excitatory drive in the network, shifting the oscillation frequency toward the higher beta range for increased levels of excitatory drive (Figures 2A, 5D). Besides affecting population firing rates and the frequency of synchronous oscillations, the excitation-inhibition balance also strongly influenced the firing pattern of STN neurons: for a low ratio of excitation to inhibition and sufficiently strong inhibitory currents, STN neurons transitioned to a firing mode characterized by low-frequency tight bursts (high intra-burst firing rate, Figures 2–4). Low-frequency bursting was periodic at 2–5 Hz but was not synchronized between cells. This shift in firing pattern toward sparse, tight bursting is in correspondence with changes in burst-related measures such as intra-burst firing rate and sub-beta band power that are most predictive of akinetic-bradykinetic symptoms in humans (Sharott et al., 2014) and monkeys (Sanders et al., 2013). The firing rate and pattern of GPe neurons was less sensitive than that of STN neurons to variations in its excitatory or inhibitory drive due to the contribution of negative feedback control by homeostatic mechanisms that operated in synergy to stabilize its E/I ratio. However, GPe neurons did synchronize more strongly under conditions of low excitatory drive from the STN enabling them to act more autonomously and synchronize through inhibitory collaterals within the GPe network.

When beta-band spiking inputs were applied to the STN-GPe network via cortico-STN afferents, the STN-GPe network phase locked to the beta rhythm. Frequencies near the autonomous oscillation frequency for a given E/I ratio were preferentially amplified, reflected in increased phase locking and power of the somatic membrane voltage at that frequency (Figure 6). This is supportive of experimental observations that oscillatory activity in STN is contingent on cortical oscillations (Magill et al., 2001), likely transmitted though the hyperdirect pathway (Tachibana et al., 2011). Phase locking and beta frequency power were further strengthened by the addition of striatal oscillatory inputs with a particular phase relationship to cortical oscillatory inputs (Figure 8). Maximum phase-locking occurred when GPe spiking was aligned in anti-phase with cortical inputs to the STN (Figures 8C,E). When excitation and inhibition occurred in anti-phase, inhibition was likely more effective at transiently hyperpolarizing the membranes of STN neurons, suggested by the local minimum in their E/I ratio (Figure 8Di). Strong hyperpolarization can evoke low-latency, temporally precise responses to an excitatory stimulus by de-inactivating Ca^2+^ and Na^+^ channels, and thereby priming them to respond to excitatory cortical inputs (Bevan et al., 2007). This mechanism may be responsible for the increase in phase locking under this phase relationship. In contrast, phase alignment of cortical and GPe neurons, corresponding to coincident firing, desynchronized STN neurons (Figure 8C). These findings are in agreement with recent experimental observations which demonstrate that co-stimulation of GABAergic and glutamergic STN afferents disperses STN spiking and has a desynchronizing effect on the population (Amadeus Steiner et al., 2019). Overall, the simulation results are consistent with the hypothesis of cortical patterning and resonance of beta activity within the STN-GPe network through feedback inhibition, whereby GPe inhibition arriving in anti-phase to cortical excitation promotes phase locking of STN neurons to beta-band cortical inputs (Baufreton et al., 2005).

### Relation of Mechanism of Oscillations to Other Models of Oscillatory Activity in the STN-GPe Network

The mechanism by which oscillatory neural activity can be generated in the STN-GPe network, by alternating phases of excitation and inhibition in a delayed negative feedback loop, has been described in previous models (Terman et al., 2002; Holgado et al., 2010; Kumar et al., 2011). The mechanism of oscillation in the model presented here is consistent with this, and the model additionally illustrates the dual role of precisely timed GPe inhibition in transiently reducing STN neuron excitability and hyperpolarizing them such that they are primed to respond with bursting to excitatory cortical inputs (Figure 9). Furthermore, it highlights the sensitivity of the network oscillation to the excitation-inhibition balance in each population and synaptic current properties.

In the multicompartment model, endogenously generated beta frequency oscillations were generated within the STN-GPe network when the strength of short duration GABA_A_-mediated currents was increased. Since the slow timescale, signaling cascade-mediated GABA_B_ currents are typically not modeled, this result can be easily reconciled with results from single-compartment and firing rate models where high gain within the closed-loop is a necessary condition for strong endogenously-generated oscillations in the STN-GPe network (Holgado et al., 2010; Park et al., 2011; Pavlides et al., 2012; Wei et al., 2015). The strength of the endogenous oscillations in our model was relatively weak, except when inhibitory GPe-STN currents were strongly dominated by fast-acting GABA_A_-mediated currents and GABA_B_-mediated slow currents were weak. The oscillation frequency of the network could be modulated by varying the ratio of excitation to inhibition in STN and GPe, and increased as this ratio increased (Figure 6).

The oscillation frequency of the network has been shown to be sensitive to model parameters in previous computational models of the BGTC network. Specifically, in mean field models of the STN-GPe loop the oscillation frequency showed a strong sensitivity to transmission delays and neuronal membrane time constants (Holgado et al., 2010; Liénard et al., 2017), and a weaker sensitivity to coupling strengths (Holgado et al., 2010; Pavlides et al., 2015; Liu et al., 2017), also demonstrated in a spiking model (Wei et al., 2015). In the multicompartment model presented here, where active ion channels on the dendrites contribute to synaptic integration, synaptic strength and effective membrane time constant are interdependent since the membrane charging speed is affected by transient activation of ion channels as a response to synaptic inputs. In biological neurons the balance of excitation and inhibition is tightly regulated through multiple adaptive processes (Turrigiano, 2011), and likely maintains the range of possible oscillation frequencies within a narrow range.

Other than the condition where GPe-STN currents were dominated by fast-acting GABA_A_ currents, strongly synchronized beta-band oscillations appeared only when exogenous beta-band inputs were introduced to the network (Figures 6, 8). These results, therefore, support a role for resonance with oscillations throughout other basal ganglia loops in the generation of increased STN-GPe beta activity in Parkinson's disease. Such an oscillatory drive can be provided either by an extrinsic oscillator, assumed to originate within the cortex in the present model, or by reverberation of oscillations in connected feedback loops such as the pallido-striatal loop (Corbit et al., 2016), intra-striatal loops (McCarthy et al., 2011), or the larger thalamocortical loop (Dovzhenok and Rubchinsky, 2012; Kang and Lowery, 2013; Pavlides et al., 2015; Reis et al., 2019). The model exhibited clear resonance in response to excitatory synaptic inputs to the STN within the beta frequency range (Figure 6). The frequency at which the maximum resonance occurred increased with increasing ratio of excitation to inhibition, similar to the increase in frequency observed in the case of endogenously generated oscillations. Resonance phenomena in the beta-band have previously been reported in computational models of basal ganglia networks, consistent with our modeling results: Pavlides et al. (2015) fitted mean field rate models to experimental data from non-human primates and found that the models that best explained the data relied on a strong cortical oscillation to sustain beta-band oscillations (~15 Hz) in the network. In a comparable mean-field model, Liu et al. (2017) found that upper beta-band (21–35 Hz) oscillations in the STN-GPe loop originated from cortical oscillatory inputs and supported a lower beta-band (12–20 Hz) oscillation that was endogenously generated. Ahn et al. (2016) using 10 single compartment STN and GPe neurons observed multiple resonances in the beta-band when varying the strength of striato-pallidal and pallida-subthalamic inhibition, with resonant peaks occurring consistently between 18 and 21 Hz. Similarly, Fountas and Shanahan (2017) found that STN neurons in their model exhibited high spontaneous beta-band power (18–30 Hz) and synchronized selectively with cortical input in this frequency range.

### Model Complexity and Limitations

One of the main advantages of the biophysically detailed model presented here is that the model can capture the non-uniform distribution of afferent inputs from different pre-synaptic populations across the dendritic tree (Tables 3, 5). This targeting of specific regions of the dendrites by different populations can lead to variations in synaptic integration properties within the structure. This feature is potentially of particular importance in the generation of pathological oscillations given that neuronal phase response curves, used to quantify the tendency of neurons to synchronize to their inputs, differ when stimuli are applied to different subcellular regions in STN and GPe neurons (Schultheiss et al., 2010; Farries and Wilson, 2012). Hence, a model that incorporates a full complement of ion channel and the synapse groups that interact with them may be expected to yield a more realistic representation of how synchronization arises in the network. In future studies, this could also contribute to a better understanding of neuronal currents contributing to the local field potential in synchronized and asynchronous states, as synaptic and ionic transmembrane currents combine to form the extracellular currents that underpin this signal (Buzsáki et al., 2012).

A second advantage of such detailed multicompartment models is that parameters have a clear relationship to the underlying biophysical system and are more meaningful in terms of physiological processes compared to models where parameters are lumped, as in single-compartment conductance-based models, or abstracted as in mean-field or generalized integrate-and-fire models. This allows for a more direct translation of experimental findings to parameter variations in the model. On the other hand, detailed cell models are more sensitive to correct estimation of these parameters which is limited by measurements performed for the purpose of model fitting as well as the fitting procedures themselves. Biophysically detailed models offer new ways to study factors contributing to the development of synchrony. Such models provide a means to investigate the relative contributions of physiological mechanisms to the development of synchrony while controlling other factors in a manner that is not possible *in vivo*. Though the model presented incorporates a higher level of physiological detail than previous models of the STN-GPe network, several simplifications were necessary due to the model complexity, which should be considered.

Downregulation of HCN channel currents with dopamine depletion was modeled as a decrease in its peak conductance. However, dopamine is known to interact with several more ion channels that are involved in linearizing the current-firing rate curve and regularizing autonomous pacemaking of STN neurons (Loucif et al., 2008; Ramanathan et al., 2008; Yang et al., 2016) which are not included in the STN cell model used here (Gillies and Willshaw, 2005). Recent evidence suggests that the loss of autonomous spiking is a necessary condition for the exaggerated cortical patterning of STN related to motor dysfunction (McIver et al., 2018). Better characterization of the ion channels involved in pacemaking and their response to dopamine depletion will enable the systematic exploration of their contribution to STN response properties and pathological firing patterns.

In our network model the main sources of firing rate variability were randomness in the input spiking patterns, the presence of surrogate Poisson spike sources in STN and GPe, membrane noise, and randomness in connection patterns and the position of synapses. However these factors do not capture the full biological variability in morpho-electric cell types, synaptic strength distributions, and resulting firing patterns in each population. In the GPe, two distinct populations have been identified based on their molecular profile and axonal connectivity (Mallet et al., 2012). Only the prototypic sub-population projecting mainly to STN and preferentially firing in anti-phase to it was modeled here, with the arkypallidal sub-populations projecting back to striatum omitted. Moreover, the GPe cell model used was only one representative candidate out of a large set of models with varying ion channel expression and morphology that matched a corresponding database of electrophysiological recordings (Gunay et al., 2008). Similarly, the STN model represents a stereotypic characterization rather than a reconstruction of a specific STN cell and does not capture variability in firing properties and receptor expression. In particular, STN neurons *in vivo* are known to have variable expression of GABA_B_ receptors (Galvan et al., 2004) which cause strong hyperpolarization responses and longer pauses in some but not all STN neurons (Hallworth and Bevan, 2005) and a strong rebound burst response (Galvan et al., 2004) in a subset of these. A model that accounts for the biological variability in GABA_B_ expression and that of channels underlying the rebound response may reveal a wider range of responses to increased inhibition among STN neurons. In such a model, beta rhythms could be transmitted to a subset of STN neurons whereas others would show longer pauses with stronger rebound bursts. Moreover, the GABA_B_ synapse model used does not fully account for activation of extrasynaptic GABA_B_R due to GABA spillover (Galvan et al., 2004) which is mediated by tonic high-frequency *and* coincident firing of afferents (Bevan et al., 2006). A model where multiple GABAergic synapses act on a shared pool of extrasynaptic GABA_B_R might increase the importance of synchronized pre-synaptic activity in switching STN neurons to a burst-firing mode.

The effect of the correlation between cortical and striatal inputs to the network was explored by varying the relative phases of both populations when firing in a synchronous oscillatory pattern (Figure 8). Uncorrelated firing between both populations was also explored (Figures 2–7). In reality, beta activity in both populations is likely to be correlated as the striatum receives topographic inputs from the same cortical areas projecting to the STN. Such correlation could lead to transient synchronization effects not explored here, that could promote or counteract additional oscillatory synchronization depending on the exact phase relationships. The effect of varying connectivity patterns between neuronal populations was not directly explored here. The development of neural synchronization and oscillatory activity are known to be dependent on network topology (Zhao et al., 2011), and this effect has previously been studied in a single compartment model of the STN-GPe network (Terman et al., 2002). The network topology used in the present study is closest to the random, sparsely-connected topology in Terman et al. (2002) which was shown to develop synchronized bursting patterns at lower frequencies. Choosing different randomly-generated connection matrices did not qualitatively change our results, however altering the connection topology would likely lead to different synchronization properties. Moreover, it is known that connection patterns within the basal ganglia are altered with dopamine depletion, particularly within the striatum (Cho et al., 2002), leading to a loss of input specificity in neuronal responses (Bronfeld and Bar-Gad, 2011). These alterations in connection patterns and resulting effects on spike correlations were not taken into account as we did not consider cortico-striatal connectivity in our model. As arkypallidal GPe neurons were not modeled, the pallido-striatal feedback loop was not captured. This additional feedback loop has also been suggested as a candidate pacemaker circuit for beta-band oscillations (Corbit et al., 2016), however, blocking of striatal inputs was not found to reduce the power of beta oscillations in rat GPe (Tachibana et al., 2011).

Finally, while there is consistent evidence of increased beta-band oscillatory activity in Parkinsons disease (Sharott et al., 2005; Mallet et al., 2008b) and a reduction of pathological beta band activity with interventions that improve symptoms in patients and animal models of the disease (Kühn et al., 2006; Weinberger et al., 2006; Ray et al., 2008; Eusebio et al., 2011), strong evidence in support of a causal role for pathological beta activity in the symptoms of Parkinsons disease has yet to be established. Indeed, recent studies failed to find evidence of any causal link between artificially induced beta band activity and motor impairment in parkinsonian rats (Swan et al., 2019), nor between the reduction of beta band activity and alleviation of motor symptoms (Pan et al., 2016). A lack of causality, however, may not necessarily be incompatible with the use of beta-band oscillations as a clinical biomarker, particularly for akinetic-bradykinetic forms of Parkinson's Disease at advanced stages of disease progression. Initial trials of adaptive or closed-loop DBS strategies targeted at suppression of beta-band activity have been successful in demonstrating simultaneous reductions in patient symptoms (Little et al., 2013; Velisar et al., 2019). Beta-band power may thus still be a suitable biomarker to indirectly gauge underlying physiological changes that are more directly related to network dysfunction such as alterations in synaptic strengths and functional connectivity within the network.

Sharott et al. (2005), Mallet et al. (2008b), and Kuhn et al. (2008), and are reduced by DBS and pharmacological interventions that alleviate parkinsonian motor symptoms (Kühn et al., 2006; Weinberger et al., 2006; Ray et al., 2008; Eusebio et al., 2011).

### Conclusion

In summary, a biophysically detailed model of the parkinsonian STN-GPe network is presented which captures non-uniform distribution of ion channels and synapses in neuronal dendrites. The network model exhibited an intrinsic susceptibility to synchronous neural oscillations within the frequency range of pathological beta-band activity observed in Parkinson's disease. Oscillations in the autonomous STN-GPe network, however, were too weak to support a pacemaker role as the sole origin of beta-band oscillations in the wider BGTC network in Parkinson's disease. In particular in the STN, autonomous beta-band oscillations and phase locking of individual cells were weak unless slower GABA_B_-mediated currents were substantially reduced. Beta-band oscillations were considerably amplified by a relatively sparse cortical beta input, with clear resonance occurring within the beta frequency range. The frequency at which the resonant peak occurred increased with increasing ratio of excitatory to inhibitory STN inputs. beta-band oscillations were further amplified by striatal beta inputs that promoted anti-phase firing of cortex and GPe. These results support the cortical patterning and network resonance hypothesis for the generation of pathological beta-band oscillatory activity in Parkinson's disease in a multi-compartment model of the STN-GPe network. They also illustrate the potential of the pallido-striatal feedback loop in further amplifying beta oscillations within the network.

## Data Availability Statement

The datasets generated for this study are available on request to the corresponding author.

## Author Contributions

All experiments were performed in the Neuromuscular Systems Laboratory in University College Dublin, Ireland. LK and ML: conceived and designed the experiments, interpreted results of experiments, prepared the figures, edited and revised the manuscript, and approved the final version of manuscript. LK: performed experiments and analyzed data and drafted the manuscript.

### Conflict of Interest

The authors declare that the research was conducted in the absence of any commercial or financial relationships that could be construed as a potential conflict of interest.
